# Ethanol alters mechanosensory habituation in *C. elegans* by way of the BK potassium channel through a novel mechanism

**DOI:** 10.1371/journal.pone.0315069

**Published:** 2025-06-11

**Authors:** Nikolas Kokan, Conny Lin, Alvaro Luna, Joani Viliunas, Catharine H. Rankin

**Affiliations:** Department of Psychology, University of British Columbia, Vancouver, British Columbia, Canada; Rosalind Franklin University of Medicine and Science Chicago Medical School, UNITED STATES OF AMERICA

## Abstract

In this research, we investigated how alcohol modulates the simplest form of learning, habituation, in *Caenorhabditis elegans*. We used our high throughput Multi-Worm Tracker to conduct a large scale study of more than 21,000 wild-type worms to assess the effects of different concentrations of alcohol on habituation of the well-characterized tap withdrawal response. We found that the effect of alcohol on habituation of this reversal response to a repeated mechanosensory stimulus (taps) differed depending on the component of the reversal response assessed. Interestingly, when we examined habituation of response probability on and off alcohol we discovered that alcohol switched the predominant response to tap from a backward reversal to a brief forward movement. Because the large conductance potassium (BK) channel has been shown to be important for the effect of alcohol on behaviour in a variety of organisms, including *C. elegans*, we investigated whether the *C. elegans* BK channel ortholog, SLO-1, mediated the effects of alcohol on habituation. We tested several different strains of worms with mutations in *slo-1* along with wild-type controls; null mutations in *slo-1* made animals resistant to alcohol induced changes in habituation. However, a mutation in the putative ethanol binding site on SLO-1 did not disrupt the impact of ethanol on habituation. Finally, by degrading SLO-1 in different parts of the nervous system we found that the function of SLO-1 in ethanol’s impact on habituation is likely distributed throughout the neural circuit that responds to tap. Based on these results, our main conclusions are 1) ethanol is not a general facilitator or inhibitor of habituation but rather a complex modulator, 2) SLO-1 is critical for the effect of ethanol on habituation, 3) ethanol is interacting (directly or indirectly) with SLO-1 through a novel unidentified mechanism to influence how animals respond to repeated taps.

## Introduction

Although alcohol alters many behavioral and cognitive functions including physical coordination, attention, and learning and memory [1,2] the effect of alcohol on the simplest form of learning, habituation, are not well understood. Habituation is defined as a decremented response to a repeated stimulus that is not due to fatigue or sensory adaptation [3]. Habituation is highly conserved across the animal kingdom and is thought to be a foundation of selective attention, which allows animals to filter out unimportant stimuli and focus on stimuli that have biological consequences. Several neuropsychiatric disorders have been associated with altered habituation (for a review see McDiarmid *et al*., [4]). For example, patients diagnosed with schizophrenia have slower habituation to sensory stimuli, which is hypothesized to be an underlying reason for their over-reactivity to stimuli [5–11]. Although the role of habituation in alcohol and substance use disorders has not received the same attention as other psychiatric disorders, there is evidence that abnormal habituation may underlie cognitive dysfunctions associated with drug addiction [12].

The existing literature on the relationship between alcohol intoxication and habituation is limited and contradictory. The first report on acute alcohol intoxication and habituation in humans investigated habituation of post-rotatory nystagmus (rapid involuntary movement of the eyes) in fighter pilots, ballet dancers, and figure skaters [13]. This study found that these subjects, known to have exceptional post-rotatory nystagmus habituation, showed no habituation when intoxicated. However, subsequent studies in humans and various other animals showed inconsistent results. Some studies found alcohol inhibited habituation (frog [14], cichlid fish [15], isolated frog spinal cord [16], mice [17]), similar to the impaired habituation of post-rotary nystagmus [13,18], while other studies found alcohol facilitated habituation (cichlid fish [15], isolated frog spinal cord [19], human [20]). Although most of the previous studies measured only a single component of behaviour, a study by Peeke *et al*. [15], found that alcohol had opposite effects on habituation of different aspects of aggressive displays in male cichlid fish. Alcohol-exposed fish showed faster habituation of response frequency, but slower habituation of response duration. These data suggest that in the same animal alcohol can have divergent effects on different components of the same response and raised the possibility that alcohol may not globally inhibit or facilitate habituation.

In this paper, we used a well-characterized genetic animal model, the 1mm long nematode *Caenorhabditis elegans*, and high-throughput behavioral analyses to investigate the effect of alcohol on habituation. A mechanical tap to the side of their Petri dish holding the worms will induce ~80% of *C. elegans* to produce a reversal, which is a brief backward movement that has been called the “tap withdrawal response” [21]. This tap withdrawal response has been well characterized ([21–25], see review [26]). When a series of taps are given at a fixed inter-stimulus interval (ISI), worms respond less and less frequently to the tap stimuli and their responses get progressively smaller. In previous studies using our high throughput machine vision Multi-Worm Tracker apparatus, we discovered that different features of the response to tap (i.e., probability, duration, and speed) can be mediated by different genes [26,27]. These data led to the conclusion that there is not a single genetic mechanism of habituation, but that habituation of different response components can be mediated by different genes. The insights from this detailed analysis of tap habituation in *C. elegans* offer an excellent opportunity to clarify the effects of ethanol on habituation.

A variety of alcohol-related behaviors have also been studied in *C. elegans* [28–43]. Ethanol has been shown to increase or decrease locomotor activity depending on the concentration of ethanol exposure [28,44,45]. Ethanol also inhibits egg-laying and increases muscle contractions when worms are on food [29,46]. More complex alcohol-affected behaviors such as state-dependent learning have also been reported in this organism [47].

A key contributor to the effect of alcohol on behavior is the BK channel, or the Big conductance voltage- and calcium-gated Potassium(K+) channel. Outside of alcohol metabolic enzymes, the BK channel was the first protein whose physiological response to ethanol was linked to the behavioral effects of ethanol in the same animal. Notably, this study was carried out using *C. elegans* and since this first association of alcohol’s effect on behavior and the BK channel [29], the effect of alcohol on BK channels has been well-documented in a variety of organisms ranging from fruit flies to mammals [48–51]. In *C. elegans*, genetic mapping of strains with strong resistance to ethanol showed that most of these strains carried mutations in the BK channel ortholog *slo-1* [29]. This resistance to ethanol was eliminated when wild-type copies of *slo-1* were restored in neurons but not in muscles [29]. Furthermore, this study found that ethanol increased the SLO-1 current by increasing the frequency of channel opening [29]. Gain of function *slo-1* mutants showed a crawling speed phenotype comparable to wild-type worms on ethanol, providing further support that hyperactivity of SLO-1 is responsible for the inhibitory effect of ethanol on crawling speed. In addition, the SLO-1 T381I substitution mutation, which is located 10–15 residues away from the SLO-1 RCK1 calcium sensor, resulted in strong resistance to the effect of ethanol on crawling speed and egg laying [52]. The T381 amino acid residue is conserved in humans, mammals and flies and is part of an ethanol binding site on the BK channel that was later identified based on amino acid substitution, electrophysiology, and crystallography data [50,52–54]. This ethanol binding site has been found to be important for *slo-1* mediated effects of ethanol on behavior in *C. elegans* [52].

The hypothesis of this study is that ethanol is not a general facilitator or inhibitor of habituation; instead, it can have different effects on habituation of different components of a behavioral response. After first validating our approach by replicating the effects of different concentrations of ethanol on body curvature, we habituated animals to taps on and off ethanol. We found that ethanol had different effects on three different components of habituation to tap: response probability, duration and speed. Furthermore, using detailed behavioral analyses we found ethanol not only affected habituation of reversal responses to repeated taps, but also switched the predominant mode of response from a reversal to a rapid forward movement. Because *slo-1* is known to be important for other behavioral effects of ethanol, we tested whether *slo-1* is also required for the effect of ethanol on habituation to repeated taps and found that the effect of ethanol on habituation was lost in *slo-1* null mutant worms. Surprisingly, the T381I region of SLO-1 that is part of an ethanol binding site on the BK channel was not important for the effect of ethanol on habituation, suggesting another site on SLO-1 is mediating its effect. By degrading SLO-1 specifically in the nervous system or the mechanosensory neurons we showed that ethanol’s effect on habituation occurred in the nervous system and not in muscles. These findings suggest that the function of SLO-1 in mediating ethanol sensitivity of response probability was most likely distributed throughout the neural circuit underlying the response to tap.

## Materials and methods

### Worm culture

*C. elegans* stocks were grown and maintained on Nematode Growth Medium (NGM) plates with the *Escherichia coli* OP50 strain as a food source [55]. N2 Bristol strain was used as the reference wild-type strain. N2, *slo-1(e.g.,142)* BZ142 (backcrossed 2X), and *slo-1(js379)* NM1968 (backcrossed 5X) strains were obtained from the *C. elegans* Genetic Center (CGC). The *slo-1(gk602291)* JPS429 (backcrossed 6X) strain was a gift from Jon Pierce-Shimomura [52]. The *slo-1(cim105[slo-1::GFP])* HKK796 *slo-1::GFP* strain and *slo-1*(*cim105*[*slo-1*::GFP]); cimSi1[*rgef-1p*::vhhGFP::*zif-1*::operon-linker::mCherry::*his-11*::*tbb-2* 3’UTR + cbr-*unc-119*(+)] HKK1165 neuron specific degraded strain were gifts from Dr. Hongkyun Kim’s laboratory [56]. The strains with touch-neuron specific degradation of *slo-1::GFP* were made from crossing HKK796 and OD2984 [ltSi953[*mec-18p*::vhhGFP4::*zif-1*::operon-linker::mKate::*his-11*::*tbb-2* 3’UTR + Cbr-*unc-119*(+)] II; *unc-119*(ed3) III] which were obtained from the CGC.

### Culture age synchronization for behavioral testing

To obtain age-synchronized colonies of animals for testing, 12.5µl of bleach reagent composed of 1:1 Bleach, 5% solution of sodium hypochlorite and 1M NaOH, as described in [57], was pipetted onto a OP50 free area of a 5 cm NGM plate seeded with OP50. A number (at least 15) of gravid adult worms were placed in the bleach dot. Adults were bleach lysed and the eggs in their gonads were released; within a few hours the larval worms hatched from the eggs and crawled to the *E. coli* [57]. The resulting colony was made up of 20–80 worms that were age matched within a range of 350 minutes, or 5–6 hours and were then used as aged-synchronized colonies [58]. These age-synchronized colonies were each grown on 5 cm NGM plates seeded with OP50 at 20^o^C for 96 hours (4 days) prior to behavioral testing.

### Ethanol treatment

Ethanol-treatment plates were prepared as follows [29,33]; prior to adding ethanol, NGM plates were seeded with *E. coli* 4 days before behavioral testing. Ethanol was then infused into the agar by pipetting 4^o^C cold 100% ethanol onto the agar to the desired concentrations based on the weight of agar in each plate using this formula: Volume = Mass x 400/ 34.26; this volume was pipetted twice to a total volume of 200–250 μL. Immediately following the addition of ethanol, plates were sealed by Parafilm, and incubated at 20^o^C for at least 2 hours or until the ethanol was absorbed and the surface of the agar was dried. Worms were transferred to ethanol plates 30min prior to the behavioral testing, and the plates were re-sealed with Parafilm.

### Behavioral recording

Behavioral recording was done using the Multi-Worm Tracker apparatus and program version 1.2.0.2 [25]. Plates containing worms were placed into the Tracker holder on the platform, and visualized using a Falcon 4M30 camera (Dalsa) and a 60mm f-number 4.0 Rodagon (Rodenstock) lens 40 cm above the platform. Images were processed using a capture card PCIe-1427 CameraLink (National Instruments). An elliptical region of interest excluding the outer 5 mm of the tracked plate was denoted on the Tracker program; this prevented tracking of animals outside of the bacterial lawn. The recording started immediately after a plate was placed on the Tracker, and worms were given 100s to acclimatize before the first tap stimulus was delivered. Taps were produced by a solenoid tapper that drives a plunger into the side of the plate at a 10s ISI. After 30 taps were delivered to the plate, worm behavior was recorded for 10s more to capture the response to the last stimulus. Each experiment was run with 3–4 plates in each group and was repeated at least 3 times.

### Body curve measurement

To assess body curve, data was extracted by the Tracker software “Choreography” as the average angle (in degrees) of the body segments for all qualified worms per frame (mean curve per frame) [25]. The mean body curve per worm was calculated from mean curve per frame during the 90s to 95s interval after the recording started (which ended 5s before the first tap stimulation). Student’s t-tests were used to evaluate differences between the average degree of body curve at different ethanol concentrations, with alpha value = 0.05.

### Tap responses analysis

Mean reversal probability, duration and speed of responses occurring within 1s of tap delivery for each plate of worms were calculated using the Choreography program [25]. A rapid forward response was defined as a response with forward bias and response velocity larger than the baseline maximum velocity 0.3s to 0.1s before a tap. Velocity was calculated by speed x bias (bias is the movement direction: 1 = forward, -1 = backward, 0 = negligible movement). Matlab® and Python scripts were used to pull and compile raw data from Choreography and then calculate rapid forward response probability from the proportion of worms that respond to a tap within 1s by briefly accelerating forward on a plate. A Pause was defined as a response with forward or reverse baseline bias and a response velocity of 0. A Reversal was defined as a response with pause or forward baseline bias and response velocity smaller than 0. The probability of a rapid forward response or a reversal response was calculated as the number of worms that had this response divided by the total number of worms that responded to the tap (excluding worms that did not respond [no change in response velocity and no change in bias]) or worms with missing data) for every 0.1 s from 0.1–0.5 s after a tap. Plates containing fewer than 10 worms responding to any tap were excluded from this calculation.

### Statistical analysis

The effects of ethanol on habituation in wild-type worms were assessed. “Initial response” was defined as the response for the first tap. “Habituated level” was calculated as the mean response to the last 3 taps (taps 28–30). Student's t-tests were used to evaluate differences in initial response and habituated level. Repeated measures ANOVAs with tap as a within subject repeated-measure factor on mean response per plate were used to evaluate the effect of independent variables on tap responses. If more than one independent variable (i.e., strain and concentration) was evaluated, then the main effects and the interactions between variables were evaluated. Tukey-Kramer posthoc tests were used to compare habituation curves between groups and to compare responses to individual taps between groups. Each sample (a plate) represents the mean value from a plate containing ~20–80 worms. “Habituation” was defined as a significant decrease in mean response level of the 28^th^-30^th^ tap compared to the initial response level, evaluated by Tukey-Kramer posthoc tests comparing the difference between the 1^st^ and 28^th^-30^th^ responses. To reduce type I error due to the large sample size (67 independent experiments consist of a total of 229 and 206 plates of worms on 0 or 400 mM ethanol, respectively), alpha was set to 0.001 instead of 0.05.

To assess the impact of genetic manipulations including *slo-1, slo-1::GFP*, or degron strains, repeated measures ANOVAs were used to determine significant effects of ethanol on reversal or rapid forward response probability. For these experiments the critical comparison was the effect of 0 or 400 mM ethanol on habituation of response probability within a strain. Tukey’s posthoc test was used to compare these groups. An ethanol group was determined to show significant ethanol effects on reversal or rapid forward response probability if Tukey’s posthoc test showed a significant difference between the 400 and 0 mM control group within a strain.

## Results

### Concentration-dependent effect of ethanol on body curve

**Fig 1:**
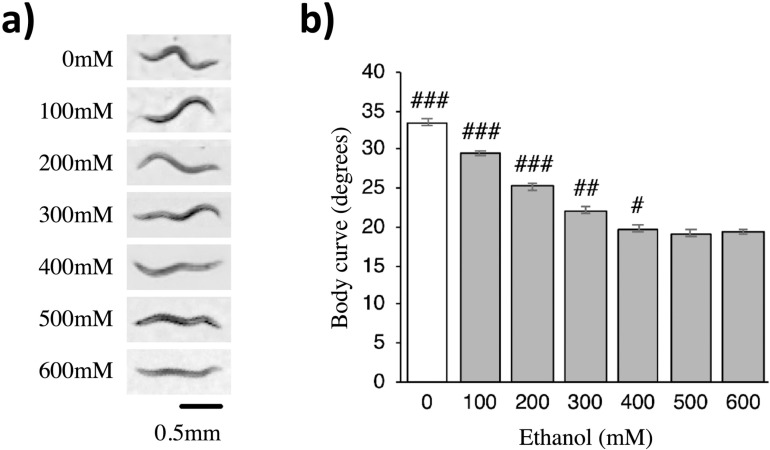
Ethanol flattens the worm’s body curve in a concentration-dependent manner. (a) Ethanol flattens worm’s body curve progressively as concentration increased from 100 to 600 mM. (b) Increasing ethanol concentrations significantly reduced the degrees of body curve of worms until the concentration reached 400 mM after which further increasing the ethanol concentration had no significant effect on worm body curve. Body curve was quantified by the Tracker software “Choreography” as the average angle (in degrees) of the tracked worms body segments. Body curve was assessed during seconds 90-95 of the experiment, just before the first tap. Error bar = SE. # = p < .05, ## = p < .001, ### = p < .0001.

To validate our protocol and ensure that our experiments replicated previously reported effects of ethanol on behavior, we assessed worm body curve because ethanol has been reported to flatten worm’s body curve in a concentration-dependent manner [29]. The ethanol treatment data collected in this study confirmed this observation (Fig 1). Worms exposed to ethanol showed flatter body curves compared to the 0 mM control (F(6)=140.68, p < 0.001, 0 mM vs 100–600 mM, all p < 0.001; Fig 1b). The curvature decreased progressively as ethanol concentration increased from 100 to 400 mM (100 mM vs 200–600 mM, p < 0.001, 200 mM vs 300–600 mM, p < 0.001, 300 mM vs 400 mM, p = 0.012, 300 mM vs 500 mM, p < 0.001, 300 mM vs 600 mM, p = 0.010). However, there were no significant differences in body curve between 400 mM and higher ethanol concentrations (400 mM vs 500 mM or 600 mM, p = n.s.). Since increasing the ethanol concentration beyond 400 mM did not produce any additional effect on body curve, later experiments in this study used 400 mM as the concentration for all ethanol treatments.

### Ethanol has different effects on habituation of response probability, duration and speed

After replicating earlier findings on body curve with our protocol, and determining that the optimal ethanol concentration to use was 400 mM, we next investigated the effects of this ethanol concentration on habituation to mechanosensory stimuli. Mechanosensory stimuli were delivered by repeatedly tapping the side of an agar filled Petri plate holding the worms mounted on the Multi-Worm Tracker [25]. To investigate how habituation of the three main components of the tap reversal response (probability, duration and speed) are affected by ethanol, we conducted a large-scale study of wild-type *C. elegans* that were given 30 repeated mechanosensory stimulations (taps) delivered at 10s ISIs on or off 400 mM ethanol.

**Fig 2:**
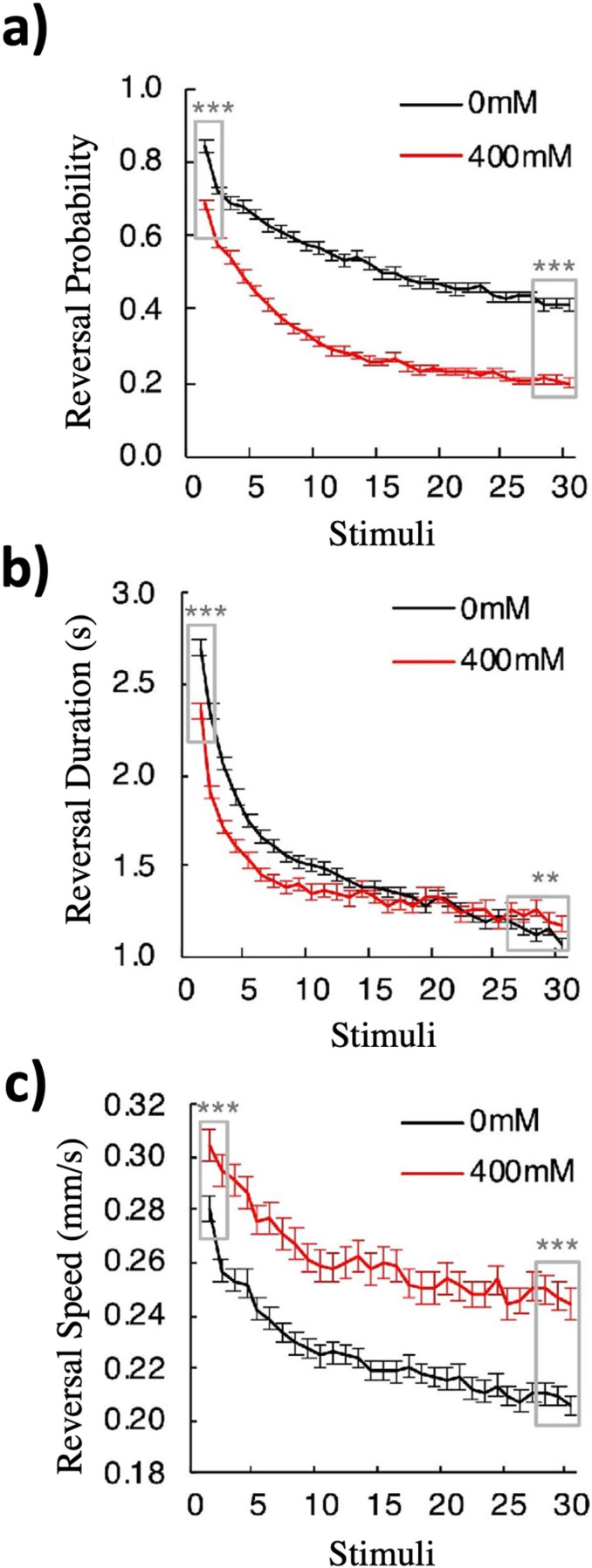
Effects of 400 mM ethanol on habituation of tap withdrawal responses in wild-type worms differ by response components. (a) Ethanol generally decreased animals’ reversal probability to tap stimuli while not having a large effect on the amount of habituation. Reversal probability was calculated as the number of worms that initiated a reversal response within 0.5s of the tap, divided by the total number of worms that responded to the tap with other responses such as pausing, accelerating, or decelerating. Boxes indicate the stimuli where statistical comparisons were made for initial and final level of habituation, using Tukey’s posthoc test. (b) Ethanol decreased the amount of habituation of reversal duration (the average time of the reversal response) and (**c**) reversal speed (the average speed of the reversal response). p < .001. Error bar = SE. * = p < .05, ** = p < .01, *** = p < .001.

Our results showed that ethanol produced different effects on the habituation of reversal probability, duration and speed (Fig 2). Firstly, the effect of ethanol on initial response level of these three habituation components was different. Initial reversal probability and duration responses of the 400 mM ethanol group were significantly reduced compared to the 0 mM control group (reversal probability: p < 0.001; duration: p < 0.001; Fig 2a and 2b), but initial reversal speed was increased compared to the control group (p < 0.001; Fig 2c). The final habituated level (the averaged mean responses to the last three taps) for reversal probability was significantly lower in the ethanol group compared to the control group (p < 0.001; Fig 2a). While the Tukey test comparing final habituated level for reversal duration between the ethanol and control groups had a low p-value (p = 0.0027; Fig 2b), this was not statistically significant because our alpha value was set at 0.001; therefore, ethanol had no significant effect on final level of duration. Interestingly, the effect of ethanol on the final habituated level of reversal speed was the opposite of its effect on reversal probability, as the ethanol group showed a higher final level compared to the control (p < 0.001; Fig 2c). Finally, the amount of habituation, as measured by the difference between the initial and final response levels, for each of the different response components was also changed by ethanol. Although reversal probability for the ethanol group appeared to habituate more than the no ethanol group (0.47 separated the initial and final levels for the 400 mM group vs 0.44 for the 0 mM group; p = 0.036; Fig 2a), this was not statistically significant due to our low alpha. However, for both duration (1.04s for the 400 mM group vs 1.53s for the 0 mM group; p < 0.001; Fig 2b) and speed (0.048 mm/s for the 400 mM group vs 0.072 mm/s for the 0 mM group; p < 0.001; Fig 2c) the ethanol group did habituate significantly less than the control group. Together, these results demonstrated that ethanol alters components of mechanosensory habituation differently in *C. elegans*. Because we observed a large effect of ethanol on both the initial and final level of habituation of response probability, and because a number of environmental factors influence response speed (e.g., temperature, humidity, wetness of the agar plates, depth of *E. coli* lawn) - some of which may be altered by adding ethanol to a plate - we chose to focus on how ethanol alters response probability for the remainder of this study.

### Ethanol changed the predominant response from a reversal to forward movement

**Fig 3:**
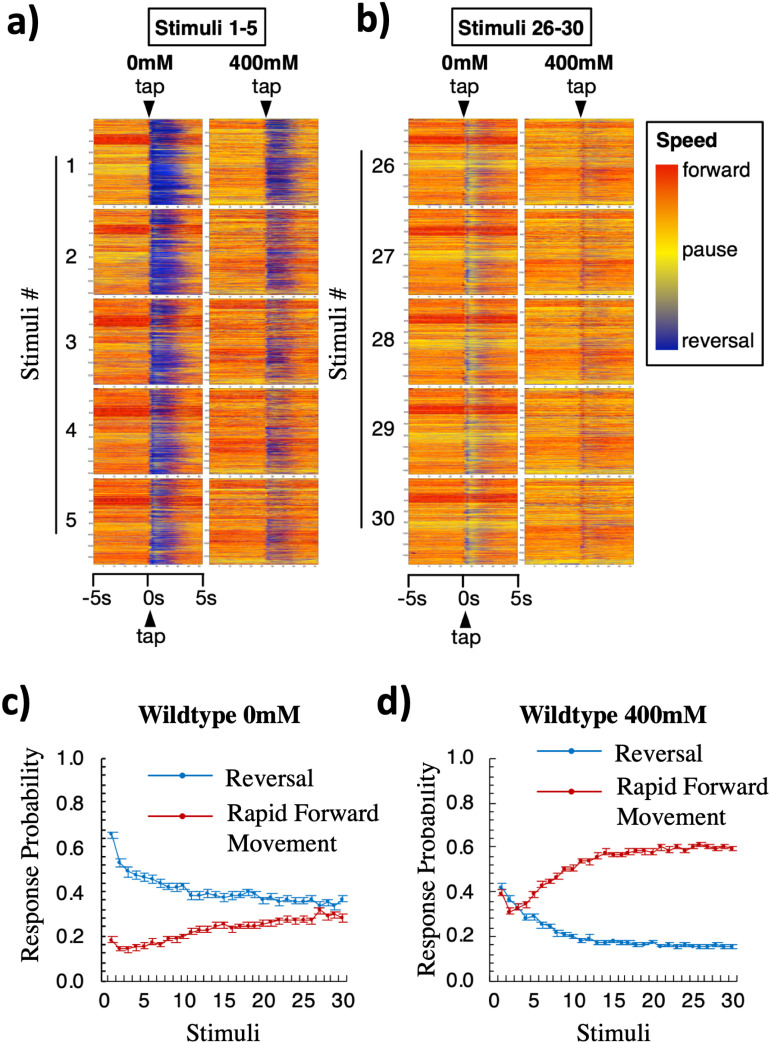
Worms on ethanol are more likely to respond to taps with rapid forward movement than a reversal. (a-b) Raster plots of velocity of locomotor movement of individual worms on 0 or 400 mM ethanol in response to the first 5 taps (a) and the last 5 taps (b) delivered at 10s ISI. Within each box, movement of an individual worm (y-axis) across time (x-axis, 5s before and 5s after a tap) is represented as a single horizontal line. The color of the line represents the direction and speed of the worm: the hotter the color, the faster the forward movement (orange to red); the colder the color, the faster the reversal movement (light to dark blue); and pauses are represented by bright yellow. Tap occurs in the middle of each box as indicated by the arrowhead at the top of each column. Data from thousands of worms were stacked vertically to construct a worm speed raster plot (each box). The numbers to the left of each pair of boxes indicates the stimulus number (1-5 or 26-30) in the 10s ISI habituation series on 0 or 400 mM ethanol plates. (c-d) Percentage of reversal or rapid forward movement response types of the total responses to taps (Stimuli) for wild-type animals (**c**) on 0 mM or (**d**) on 400 mM ethanol. Reversal/rapid forward movement probability was calculated as the number of worms that initiated a reversal/rapid forward movement response within 0.5s of the tap, divided by the total number of worms that responded to the tap. Errorbar = SE.

Although a reversal is the predominant response to taps for wild-type worms, it is not the worm’s only mode of response to taps [59,60]. Worms can also respond to taps by accelerating, pausing, or decelerating. To examine these dynamic responses to taps, changes in speed and direction of movement over time were visualized using direction and speed-based raster plots (Fig 3a). For the first tap (Stimulus 1), the raster plots for most of the control group show blue horizontal lines starting from the delivery of the tap stimulus. This corresponds to worms responding to the first tap by reversing and moving backwards for several seconds. Compared to worms on the control plates, the raster plot for the ethanol group shows less blue; this corresponds to the observation that ~25% fewer worms on ethanol reversed in response to the first tap (Fig 3). From Stimuli 1–5, both the ethanol and control groups showed progressively fewer reversals in response to each stimulus, and fewer worms in the ethanol group reversed in response to taps by stimulus 5 (Fig 3a). Raster plots for the last 5 stimuli (Stimuli 26–30) showed shorter and slower reversals and fewer worms responding to the tap in both groups (Fig 3b).

When the raster plots for Stimuli 26–30 were examined closely, the response pattern of worms in the ethanol group shows a thin vertical red band at the time of tap delivery (Fig 3b). This indicates that to the majority of the worms on ethanol responded to the final stimulus by moving rapidly forward (red) for a very short period of time. In contrast, raster plots for Stimuli 26–30 for the no alcohol group show a thin vertical blue band shortly after the tap was given followed by a wider light yellow vertical band, indicating that instead of long reversals, many worms in the control group responded to the last 5 taps by reversing very briefly then pausing (yellow) for ~1–2 s. Additionally, some worms simply paused for a few seconds rather than reversing. These data suggest that ethanol does not simply decrease the reversal probability to repeated taps as shown in (Fig 2a), but switched the predominant response mode from a reversal to forward movement.

To quantify this switch in the predominant response mode, the probability of reversal or rapid forward responses to each tap were plotted in Fig 3c and 3d. For both groups, the probability of a reversal response decreased as the number of taps increased, similar to data shown in (Fig 2a). In contrast, the probability of rapid forward responses gradually increased as the number of taps increased for both groups (Repeated measures ANOVA: F(58,14268)(response mode)=54.083, p < .001; Fig 3c and 3d). For both groups, the ethanol treatment had a significant effect on response mode probability (F(24,14628)(concentration)=2.33, p < .001, F(58,14268)(response mode*concentration)=3.16, p < .001). For worms in the no alcohol group, from the first to last tap the probability of a reversal response was always higher than the probability of a rapid forward response (Fig 3c). Although the probability of reversal and rapid forward response approached each other during the final taps, at no point was the probability of a rapid forward movement higher than that of reversals (rapid forward vs. reversal responses, tap 22, 27, 29, p = n.s., all other taps, p < .001). In contrast, worms in the ethanol group initially had similar reversal and rapid forward response probabilities (rapid forward vs. reversal responses, tap 1–4, p = n.s.), but after the 4^th^ tap, the rapid forward response probability became significantly higher than reversal response probability (rapid forward vs. reversal responses, tap 5–30, p* *< .001; Fig 3d). These data indicate that ethanol not only altered habituation of reversal responses to taps, but also changed the predominant tap response from a reversal to a rapid forward movement.

### The BK channel ortholog slo-1 mediates the effect of ethanol on tap response mode

**Fig 4:**
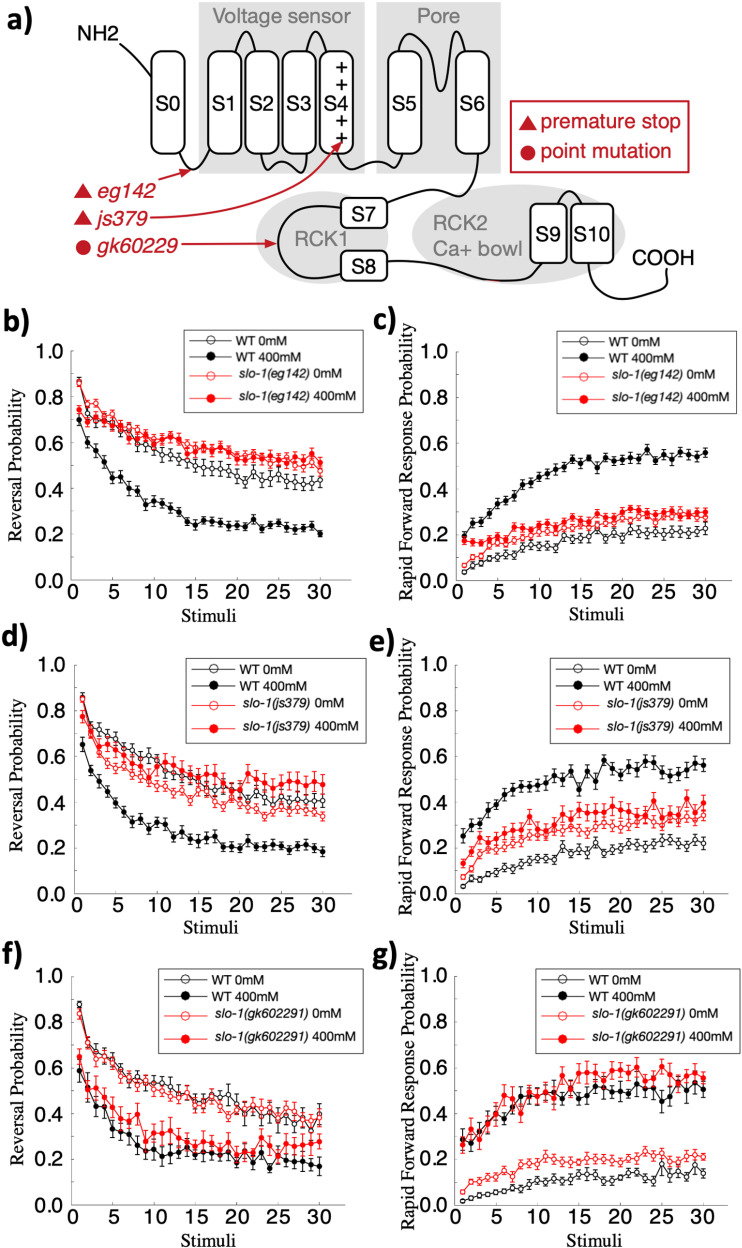
*slo-1* null mutations eliminate the effect of 400 mM ethanol on reversal and rapid forward response probability while a point mutation in the putative ethanol binding in RCK1 retains the ethanol mediated effect. (**a**) SLO-1 protein structure indicating the locations of three alleles tested by red arrows. *slo-1(e.g.,142)* contains a nonsense mutation at the first transmembrane motif S0. *slo-1(js379)* contains a nonsense mutation at the ion pore voltage sensing region. *slo-1(gk602291)* contains a T381I point mutation in the putative ethanol binding region. This part of the figure is a modified from a figure in [66]. (**b**) Ethanol did not impact the reversal and (**c**) rapid forward response probability of worms carrying the *slo-1(e.g.,142)* null allele. Reversal/rapid forward response probability was calculated as the number of worms that initiated a reversal/rapid forward response within 0.5s of the tap, divided by the total number of worms that responded to the tap. (**d**) The effect of ethanol on worms carrying the *slo-1(js379)* null allele is inverted and reduced for reversal probability (**e**) and is lost entirely for rapid forward response probability. (**f**) The effect of ethanol on worms carrying the *slo-1(gk602291)* allele is the same as on wild-type animals for reversal (**g**) and rapid forward response probability. Errorbar = SE.

The BK channel is a well characterized target of ethanol in mammals and in *C. elegans* [29,52,61–65]. The BK channel is composed of four pore-forming alpha subunits (Fig 4a). Each alpha subunit consists of seven transmembrane domains (S0-S6) and long intracellular hydrophobic segments (S7-10) [66]. Within the transmembrane domains, the voltage sensor (S1-4) detects membrane potential changes and the pore-forming domain (S5-6) allows passage of potassium ions. The long intracellular hydrophobic segments (S7-10) contain two main calcium sensors, the RCK1 and RCK2, that allow calcium-coupled channel activation and also contains a putative ethanol binding domain near RCK1 that is thought to be crucial for *slo-1*’s interaction with ethanol [53,65]. In *C. elegans*, mutations in this ethanol binding region of *slo-1* block the inhibitory effect of ethanol on crawling speed and egg laying [52]. Therefore, we hypothesized that ethanol would have less of an effect on habituation in a *slo-1* mutant with a mutation in this ethanol binding site.

To investigate whether the BK channel is involved in the ethanol induced changes in tap response habituation, we tested mutant strains carrying three different *slo-1* alleles (Fig 4a). Two of the three alleles, *e.g.,142* and *js379*, are putatively null alleles [29,67] with a nonsense mutation at the first transmembrane motif S0 [66], or at the ion pore voltage sensing region, respectively. The other allele, *gk602291,* has a point mutation in the ethanol binding region near the calcium sensing domain RCK1. This point mutation substitutes a highly conserved threonine to isoleucine at position 381 of the SLO-1 channel (T381I) [52]. *slo-1(gk602291)* mutants have been shown to have strong resistance to the inhibition of locomotor activity and egg laying by ethanol, similar to that of null mutant *slo-1(js379)*, but unlike in the null mutant, basal behaviors such as neck posture (body curve measure of the anterior part of the animal) were unaltered by this mutation [52].

Plates of each of the three *slo-1* mutant strains and wild-type worms were given 30 tap stimuli at a 10s ISI in the presence of either 0 mM or 400 mM ethanol. The wild type worms showed large differences for both reversals and rapid forward response when worms in the 0 mM groups were compared to the 400 mM groups. In contrast, the 0 and 400 mM groups of worms carrying either of the two null alleles of *slo-1* were very similar for habituation of both reversal or rapid forward movement probability, and both null mutant groups looked similar to the 0 mM wild-type worms (Fig 4b–4e). A repeated measures ANOVA comparing wild-type worms to worms carrying the null allele *slo-1(e.g.,142)*, given 30 taps on and off of ethanol, replicated the original observation that ethanol reduced reversal probability and increased rapid forward response probability in wild-type worms in response to taps (rmANOVA(reversal): F(29,4814)(strain*ethanol*tap)=2.835, p < .001, posthoc: wild-type vs. wild-type ethanol, p < .001; rmANOVA(forward): F(29,4843)(strain*ethanol*tap)=5.934, p < .001, posthoc: wild-type vs. wild-type ethanol, p < 0.001), while the presence of ethanol failed to show a significant effect on the reversal or forward movement probability of *slo-1(e.g.,142)* worms (posthoc: *slo-1(e.g.,142)* vs. *slo-1(e.g.,142*) ethanol, p = n.s., for both reversal and rapid forward responses; Fig 4b and 4c). This indicates that the *slo-1(e.g.,142)* null mutation greatly reduced or eliminated the effect of 400 mM ethanol on both reversal and rapid forward response probability to repeated taps. For the second putative null allele *slo-1(js379)*, the repeated measures ANOVA showed a significant effect of ethanol on reversal probability (rmANOVA: F(29,4901)(strain*concentration*tap)=2.51, p < .001, posthoc: *slo-1(js379)* vs *slo-1(js379)* ethanol, p = .03; Fig 4d), and failed to show a significant effect of ethanol on rapid forward response probability (posthoc: *slo-1(js379)* vs *slo-1(js379)* ethanol, p = n.s.; Fig 4e). Interestingly, the effect of ethanol on reversal probability was opposite to that of wild-type, where *slo-1(js379)* mutants showed a slight increase in reversal probability (Fig 4d). Importantly, posthoc comparison showed that the reversal probability habituation curve of *slo-1(js379)* mutants for the ethanol or no ethanol groups were not significantly different from that of wild-type, indicating that ethanol had much less of an effect on reversal probability response of the *slo-1(js379)* mutants than on wild-type worms (posthoc: wild-type vs *slo-1(js379)* and wild-type vs *slo-1(js379)* ethanol, p = n.s.). These findings are very similar to the findings from the other null mutation, *slo-1(e.g.,142)*, supporting the hypothesis that *slo-1* plays an important role in how ethanol alters habituation of behavioral responses to repeated stimuli.

Worms carrying the *slo-1(gk602291)* allele with a mutation in the ethanol binding domain have been reported to show similar resistance to the inhibitory effect of ethanol on egg laying and locomotor activity as worms carrying the null allele *slo-1(js379)* [52]. However, we observed no significant differences in habituation of reversal and rapid forward response probability between *slo-1(gk602291)* mutants and wild-type worms (rmANOVA(reversal): F(29,1450)(strain*concentration*tap)=0.88, p = n.s.; posthoc: *slo-1(gk602291)* vs *slo-1(gk602291)* ethanol, p=<0.001; wild-type vs wild-type, p=<0.001; Fig 4f and 4g). To confirm the presence of the *gk602291* allele we sequenced the JPS429 strain twice, once when we initially ran the experiments, and again before submission of this manuscript. Like the *slo-1* null mutations, this allele did confer resistance to the effect of 400 mM ethanol on body curve which aligns with past research showing that other ethanol mediated behaviors can still be impacted by this mutation (Fig S1 in S1 File) [52]. The data reported here suggests that the *slo-1* T381 residue in the ethanol binding domain is not involved in ethanol’s effect on habituation of reversal or rapid forward response probability to repeated taps.

### Cell-specific degradation of the BK channel revealed slo-1 expression in neurons is responsible for the effect of ethanol on response mode

The effects of ethanol on habituation might be mediated by the effect of ethanol on the whole animal, on the nervous system, on the muscles or on a specific subset of neurons*. slo-1* is known to be expressed in both neurons and muscles. To investigate which cells are responsible for the role of *slo-1* in mediating the effects of ethanol on habituation, SLO-1 was selectively degraded in all neurons, or specifically in mechanosensory neurons (Figs S2–S5 in S1 File) [68]. To do this, worms with GFP inserted into *slo-1*’s C-terminus were crossed into degron strains that drove degradation specifically pan-neuronally (*rgef-1p*) or in the mechanosensory neurons (*mec-18p*).

**Fig 5:**
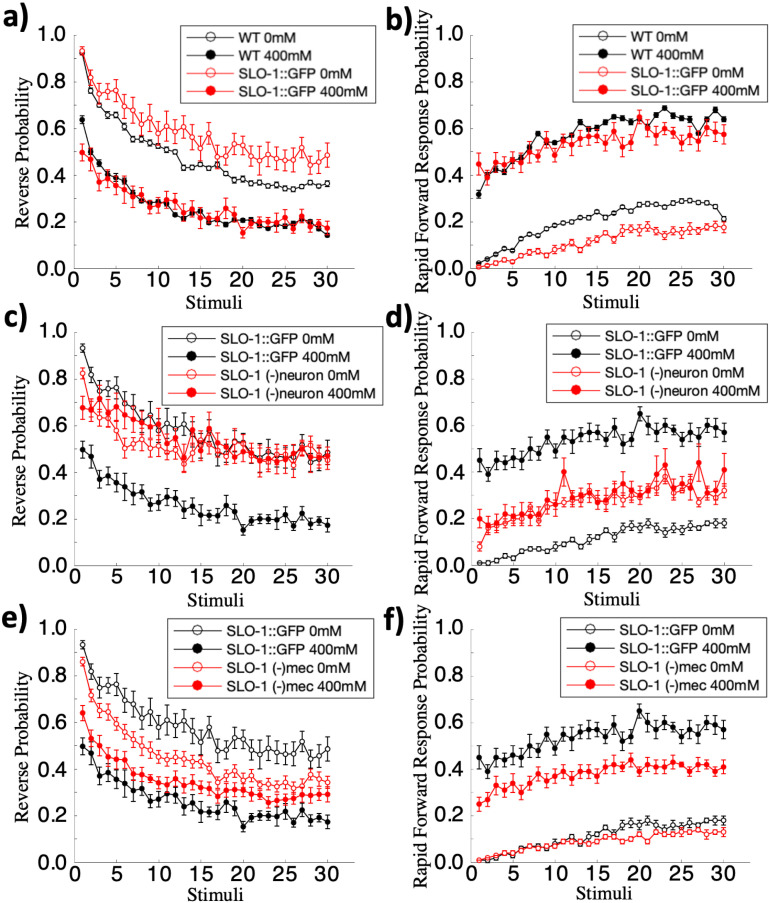
Cell-specific SLO-1 degradation indicates SLO-1 expression in neurons is required for the effects of ethanol on reversal and rapid forward response probability. (**a**) Ethanol similarly decreased reversal probability, and (**b**) increased rapid forward response probability in worms expressing *SLO-1::GFP* as it does in wild-type worms. Reversal/rapid forward response probability was calculated as the number of worms that initiated a reversal/rapid forward response within 0.5s of the tap, divided by the total number of worms that responded to the tap. (**c**) The effects of ethanol on reversal probability are lost in worms with SLO-1 degraded in neurons (SLO-1 (-)neuron), (**e**) and dampened in worms with SLO-1 degraded in mechanosensory neurons (SLO-1 (-)mec). In these experiments, SLO-1::GFP was degraded by selective expression of a GFP binding nanobody that induces degradation [68]. (**d**) Similarly, the effects of ethanol on rapid forward response probability are lost in worms with SLO-1 degraded in neurons (SLO-1 (-)neuron) (**f**) and in worms with SLO-1 degraded in mechanosensory neurons (SLO-1 (-)mec). Errorbar = SE.

As a control we first showed that the GFP insertion did not greatly disrupt *slo-1* function during habituation. While there were minor difference between the *slo-1::GFP* strain and wild-type worms (rmANOVA(reversal): F(29,7192)(strain*tap)=2.82, p= < 0.001; posthoc: *slo-1::GFP* vs wild-type, p=<0.001), critically ethanol has a significant effect on both reversal and forward movement response probability of *slo-1::GFP* worms (posthoc: *slo-1::GFP* vs *slo-1::GFP* ethanol, p=< 0.001; Fig 5a and 5b). These results suggest that these *slo-1::GFP* worms may have slightly altered baseline habituation responses to repeated taps because of the GFP addition onto SLO-1, or because of differences in the background of the *slo-1::GFP* strain and our wild-type controls. However, these data showed that ethanol’s effect on reversal and rapid forward response probability in *slo-1::GFP* worms is intact in these worms. Moreover, expression of the GFP degron pan-neuronally or in the mechanosensory neurons does not alter the effect of ethanol on reversal and rapid forward response probability (Fig S6 in S1 File).

To test whether *slo-1* expression in neurons is responsible for the effect of ethanol on reversal and rapid forward response probability, SLO-1::GFP worms were crossed into a degron strain that targeted degradation of SLO-1 pan-neuronally (SLO-1(-)neuron) and these worms were habituated on and off of ethanol. Results showed that worms without SLO-1 in their neurons failed to show an effect of ethanol on reversal or rapid forward response probability (posthoc(reversal): SLO-1(-)neuron vs SLO-1(-)neuron ethanol, p = n.s.; posthoc(forward): SLO-1(-)neuron vs SLO-1(-)neuron ethanol, p = n.s.; Fig 5c and 5d). This indicates that *slo-1* expressions in neurons is required for ethanol to affect the reversal and rapid forward response probability.

When SLO-1::GFP was degraded in just the mechanosensory neurons, the ethanol group still showed a significant decrease in reversal probability (posthoc: SLO-1(-)mec vs SLO-1(-)mec ethanol, p = .021; Fig 5e and 5f). However, ethanol effects were smaller than the effects observed in wild-type worms. Similarly, although ethanol did effect rapid forward response probability of SLO-1(-)mec worms (SLO-1(-)mec vs SLO-1(-)mec ethanol, p < .001), and SLO-1(-)mec worms displayed similar rapid forward response probability to the *slo-1::GFP* control when not on ethanol (posthoc: SLO-1(-)mec vs *slo-1::GFP*, p = n.s.), when exposed to ethanol SLO-1(-)mec worms had significantly different rapid forward response probability curves compared to the *slo-1::GFP* control (posthoc: SLO-1(-)mec ethanol vs *slo-1::GFP* ethanol, p < .001). This suggests that while SLO-1 in mechanosensory neurons plays some role in mediating the effect of ethanol on reversal and rapid forward response probability, SLO-1 in other neurons is likely playing a role as well.

## Discussion

This study used *C. elegans* to examine the effects of ethanol on mechanosensory habituation. We found that ethanol had divergent effects on different response components (probability, duration, and speed), and on different modes of responses (reversal vs. rapid forward movement). Exposure to ethanol resulted in a decrease in the initial response for reversal probability and duration but increased it for reversal speed. Ethanol also decreased the final level of response after tap habituation for reversal probability and increased it for reversal speed. Additionally, ethanol decreased the amount of habituation observed for reversal duration and reversal speed and had little effect on the amount of habituation of reversal probability; the ethanol group actually displayed a non-significant increase in habituation of reversal probability. However, while exposure to ethanol did result in a general decrease in reversal probability to the tap stimuli, it simultaneously produced an increase in the probability of a rapid forward response compared to worms in the ethanol free condition. The BK potassium channel SLO-1 was critical for ethanol’s effects on habituation of response probability as null alleles of this gene eliminated the impact of 400 mM ethanol’s on habituation to taps. Interestingly, a mutation in an ethanol binding domain near RCK1 did not disrupt the effect of ethanol on habituation of response probability, leading to the hypothesis that this binding site is likely not involved in the effect of ethanol on this aspect of behavior. Preliminary investigations into where SLO-1 is needed for its effect on habituation suggested ethanol affects habituation of response probability through *slo-1* expression in neurons. As degrading SLO-1 in mechanosensory neurons attenuated, but did not eliminate, the effect of ethanol on habituation, SLO-1’s function is probably distributed across the neural circuit that controls mechanosensory responses.

### Ethanol can both facilitate and inhibit habituation

From the first report on the effect of alcohol on habituation in 1967 [13], there has been an ongoing debate about whether alcohol inhibits or facilitates habituation [13–20,69,70]. Prior to our study, six publications support an inhibitory effect of alcohol on habituation [13,14,16–18,69], and three publications support a facilitative effect [19,70,71]. Only one report encouraged the field to consider a more complex effect of alcohol on habituation than a straight-forward inhibitory or facilitatory role [15]. Peeke *et al*. [15] found 0.3% ethanol facilitated habituation of aggressive display frequency towards conspecific male cichlid fish, but inhibited habituation of aggressive display duration [15]. However, perhaps due to small sample size (N = 7), the authors of this study [15] did not make a strong claim about the bi-directional effects of alcohol on habituation. Although alcohol has since been known to have bi-directional effects on other types of behaviors, there was no strong evidence demonstrating ethanol could have opposite effects on habituation within the same response. Our data, based on a large dataset with high statistical power (77 experiments containing more than 21,000 animals), provides the first convincing data supporting the hypothesis that alcohol can have divergent effects on habituation of different components within the same response (Fig 2). This work suggests that it is not useful to think of alcohol as a general inhibitor or facilitator of habituation, but rather as a complex modulator of habituation. This would explain why previous findings on the impact of ethanol on habituation were contradictory, as these studies typically scored habituation of a single component of a response.

However, there are two potential confounds that should be addressed. First, ethanol at this concentration is known to decrease basal locomotion speed, so it is possible that this suppression of basal locomotion speed could cause suppression of worms ability to respond to the tap stimulus [29,52]. However, basal speed has been found to correlate poorly or not at all with the initial reversal probability, reversal duration and reversal speed in response to tap, and the habituation of these responses [27]. This suggests that basal locomotion speed and these response metrics rely on distinct processes and that a factor affecting one will likely not impact the other. Additionally, if ethanol’s suppression of basal locomotion speed did affect the ability to respond to tap, we would expect a suppression of the animal’s naïve response to the initial stimulus. This was not observed for reversal speed or reversal probability. For reversal speed, the initial reversal speed was actually significantly higher in the 400 mM ethanol group than the no ethanol group. For reversal probability, while the initial reversal probability is significantly lower in the 400 mM ethanol group, there is a corresponding increase in a different locomotor response: rapid forward movement. Reversal duration does display a non-significantly reduced initial response in the 400 mM ethanol group. Therefore, it is possible that suppressed basal locomotion could be impacting our reversal duration measure, however since the reversal speed increased in the ethanol group it would be surprising that suppressed basal locomotion speed would selectively impact reversal duration.

A second variable that is potentially impacting our results is acute tolerance to ethanol. After 30 minutes, worms have built up some tolerance to the effects of ethanol, including on locomotion [30,72]. Therefore, it is possible that the effect of ethanol we observe on the tap reversal response metrics we assessed (probability, duration and speed) might have been larger if assessed sooner after ethanol exposure. Since there is less than 5 minutes between the 1^st^ and 30^th^ tap stimulus, any change in response due to an increase in ethanol tolerance during the experiment is likely minimal.

### Ethanol exposure altered response mode from reversals to rapid forward movement

Another significant discovery of this study is that alcohol altered response propensity from a reversal to forward movement (Fig 3). The significance of this finding can be better appreciated once given more background on the natural adaptive behavior in worms. The directionality of worm movement in response to environmental cues can be generally divided into decisions between “avoid” or “approach” (Reviewed in [73]). When given noxious stimuli, such as heat, light, noxious chemicals, high osmolality, or a nose/head touch, worms avoid the stimuli by moving in a direction opposite to the source of the stimuli. Tap stimuli, on the other hand, produce vibrations without a clear direction of the source. In this case, adult worm’s neuronal circuits are biased to produce reversals followed by a change in direction and then forward movement [74–76], suggesting that a reversal followed by a turn is an adaptive response when the direction of the noxious stimuli isn’t clear. In contrast, worms move forward from a tail touch or a heat stimulus to the tail, or to a noxious compound behind the animal; this is most often a forward movement lasting at least several seconds. The brief forward movement we observed in this study does not resemble the natural “approach” behavior. Qualitatively, during approach behavior worms crawl towards the attractive cue, which is accomplished by inhibiting reversals and steering towards the source of the cue [73]. This is very different from the brief, short forward movement observed in worms on ethanol in response to tap. These forward responses were as rapid as the reversal responses (both can achieve a speed of 0.3–6 mm/s), and are two times faster than the exploratory/approaching behavior (typically between of 0.1–0.3mm/s) [77]. This suggests that the forward response to taps, just like the reversal to taps, might appropriately be described as a “startle” behavior, or could be classified as an “avoid” behavior from the Faumont review [73].

We found that worms on alcohol were equally likely to move rapidly forward or to reverse in response to the first tap, while worms without alcohol were about three times more likely to reverse than to move forward (Fig 3). This difference suggests that alcohol disturbed the innate bias to reverse and then move forward in a different direction, when exposed to a stimulus without a clear location. Strikingly, after only a few taps, worms on alcohol became more likely to move forward than to reverse, which never occurred throughout all 30 taps for the control worms not exposed to ethanol. By the 15^th^ tap, worms on alcohol were 3 times more likely to move forward than to reverse. This suggests alcohol produced a behavioral bias opposing the propensity to reverse in response to a startling non-localized stimulus. Previous studies hypothesized that the direction of a worm’s tap response results from the balance of strength between the forward and backward circuit [74,78]. Ablation of neurons in the forward circuit increased reversal probability, and ablation of neurons in the backward circuit increased rapid forward response probability and decreased reversal probability [79]. One possibility is that the switch to higher forward movement probability observed in worms on ethanol came from selective inhibition of the backward circuit and/or enhancement of the forward circuit. This altered response bias represents a novel opportunity to further our understanding on alcohol’s effect on behavior.

### The BK channel SLO-1 is necessary for the effects of 400 mM ethanol on habituation of reversal and rapid forward response probability, however the known ethanol binding site on SLO-1 is not required

This study provides evidence that the BK channel is important in the effects of alcohol on habituation of worm’s response to tap stimuli. However, the BK channel’s known ethanol binding domain near RCK1 was not important in mediating this effect on habituation, which is a stark difference from what has been described for other behaviors.

The BK channel’s role in mediating the effects of ethanol on behavior has been well established in *C. elegans* research [29]. In fact, *C. elegans* provided the first evidence linking the physiological effect of ethanol on ion channels with its effects on behavior [29]. Following that, an ethanol binding site close to the BK channel’s intracellular RCK1 domain was identified [50,53]. The BK channel’s role in ethanol modulated behavior in *C. elegans* has since been well characterized, including inhibition of locomotor, egg laying [29], inhibition of pharyngeal activities [64], and a role in acute ethanol tolerance [63]. The work reported here demonstrated that SLO-1 also plays a crucial role in ethanol-induced changes to responses to repeated stimuli. In the absence of ethanol, we found no evidence of a role for SLO-1 in altering response probability after repeated taps (Fig 4b–4e). However, null mutations in *slo-1* eliminated the effects of 400 mM ethanol on habituation of reversal and rapid forward response probability (Fig 4b–4e), indicating that the BK channel may be necessary for ethanol to alter response mode. While we tested *slo-1* mutants at 400 mM ethanol, it is possible that even greater concentrations of ethanol could produce a significant effect on habituation of response probability in *slo-1* null mutants.

We also tested animals with the T381I mutation near the RCK1 calcium sensing domain of SLO-1, which substitutes a highly conserved amino acid. Despite being very close to the RCK1 calcium sensing region of SLO-1, it has been hypothesized that the T381 amino acid might be part of a conserved ethanol binding/activation site on SLO-1 as most of the 20 closest amino acid residues are conserved and mutations in T381 cause ethanol insensitivity in worm and mammalian SLO-1 channels [52]. Furthermore, the same conserved location on mammalian SLO-1 has been identified as an ethanol binding site and T381 is thought to be structurally important for ethanol binding [50,53,65]. It has been shown that ethanol binding at this site facilitates the natural interaction with calcium that gates the SLO-1 channel and is necessary for ethanol induced increases in SLO-1 channel activity [50,53]. Previous research found that the T381I mutation played an important role in the effects of ethanol on behavior, making locomotion and egg laying behaviors resistant to inhibition by ethanol, similar to a *slo-1* null mutant [52]. Notably, the SLO-1 channel in T381I mutants is still functional, and for ethanol independent behaviors that were impacted by *slo-1* null mutants, such as neck posture, T381I animals exhibited wild-type behavior [52]. Interestingly, we found that the T381I mutation did not impact the effect of ethanol on habituation (Fig 4f and 4g). This suggests that another site on the SLO-1 channel mediates the effect of alcohol on habituation and that the T381I region is not required for all of ethanol’s behavioral modulations. Therefore, different domains of the BK channel may mediate the effects of ethanol on different behaviors. While there might be another site on the BK channel that ethanol directly interacts with, SLO-1 activity can also be regulated by numerous other mechanisms including calcium influx, alpha subunit alternative splicing, modulation by kinases, and regulation by auxiliary beta subunits, [53,65,80]. Further investigation is necessary to confirm that other mutations in this ethanol binding site also fail to stop the impact of ethanol on response mode, and to elucidate which other SLO-1 regulatory mechanisms or additional ethanol binding sites are required for ethanol effect on mechanosensory habituation response mode.

### The effect of ethanol on response mode to repeated mechanosensory stimuli is likely mediated by *slo-1* expression in neurons

*slo-1* is widely expressed in neurons and muscles and acts in muscles at the neuromuscular junction [66]. By degrading SLO-1 in neurons, we showed that SLO-1 action in neurons is critical for the effect of ethanol on response mode to repeated mechanosensory stimuli (Fig 5c and 5d). We further investigated whether the effect of ethanol on response probability was mediated by *slo-1* expressed specifically in the mechanosensory neurons that respond to the tap. The tap withdrawal neuronal circuit is composed of 5 mechanosensory neurons (ALML/R, PLML/R, and AVM), 5 pairs of premotor interneurons (AVAL/R, AVBL/R, AVDL/R, PVCL/R, and RIM), a pair of harsh touch neurons PVDL/R, and a single proprioception neuron DVA [78,79,81]. We selectively degraded SLO-1 in mechanosensory neurons and found that SLO-1 in mechanosensory neurons is only partially responsible for ethanol altering habituation of reversal and rapid forward response probability (Fig 5e and 5f). Given these findings, it is probable that the main sites of ethanol’s action are distributed over multiple neurons of the tap withdrawal circuit.

## Conclusion

In this study, we demonstrated a complex effect of ethanol on different response components of habituation to repeated mechanosensory stimuli. The data suggests that ethanol acts as a complex modulator of habituation and explains why earlier work found both faciliatory and inhibitory effects of ethanol on habituation. We also identified a shift in behavioral response bias as a result of ethanol intoxication. These findings promote the use of a more detailed characterization of habituation when investigating the effect of ethanol. Exclusively observing how ethanol impacts a single response component might miss important differences in other response components and lead to incomplete conclusions.

We found that the SLO-1 BK channel was required in the nervous system for alteration of habituation of response probability by ethanol, although SLO-1’s known ethanol binding site was found to not be necessary for modulation of habituation of response probability by ethanol. Future investigation into how ethanol interacts with SLO-1 to mediate changes in response probability could identify a novel ethanol binding site on SLO-1 or an ethanol mediated indirect signaling pathway, advancing our understanding of the complex interactions between alcohol and the nervous system.

## Supporting information

S1 File S1-S6 Fig. Supplemental Figures. This file contains supplementary figures S1-S6 as well as the figure captions for S1-S6. (ZIP)

## References

[1] Leckliter IN, Matarazzo JD. The influence of age, education, IQ, gender, and alcohol abuse on Halstead-Reitan Neuropsychological Test Battery performance. J Clin Psychol. 1989;45(4):484–512. doi: 10.1002/1097-4679(198907)45:4<484::aid-jclp2270450402>3.0.co;2-l 2671047

[2] Selby MJ, Azrin RL. Neuropsychological functioning in drug abusers. Drug Alcohol Depend. 1998;50(1):39–45. 9589271 10.1016/s0376-8716(98)00002-7

[3] Thompson RF, Spencer WA. Habituation: a model phenomenon for the study of neuronal substrates of behavior. Psychol Rev. 1966;73(1):16–43. 5324565 10.1037/h0022681

[4] McDiarmid TA, Bernardos AC, Rankin CH. Habituation is altered in neuropsychiatric disorders—A comprehensive review with recommendations for experimental design and analysis. Neurosci Biobehav Rev. 2017;80:286–305. 28579490 10.1016/j.neubiorev.2017.05.028

[5] Venables PH. Psychophysiological aspects of schizophrenia. Br J Med Psychol. 1966;39(4):289–97.

[6] Depue RA, Fowles DC. Electrodermal activity as an index of arousal in schizophrenics. Psychol Bull. 1973;79(4):233–8. doi: 10.1037/h0034010 4699784

[7] Gruzelier J, Eves F, Connolly J, Hirsch S. Orienting, habituation, sensitisation, and dishabituation in the electrodermal system of consecutive, drug free, admissions for schizophrenia. Biol Psychol. 1981;12(2–3):187–209. doi: 10.1016/0301-0511(81)90011-9 7332772

[8] Geyer MA, Braff DL. Habituation of the Blink reflex in normals and schizophrenic patients. Psychophysiology. 1982;19(1):1–6. doi: 10.1111/j.1469-8986.1982.tb02589.x 7058230

[9] Braff DL, Grillon C, Geyer MA. Gating and habituation of the startle reflex in schizophrenic patients. Arch Gen Psychiatry. 1992;49(3):206–15. doi: 10.1001/archpsyc.1992.01820030038005 1567275

[10] Hollister JM, Mednick SA, Brennan P, Cannon TD. Impaired autonomic nervous system-habituation in those at genetic risk for schizophrenia. Arch Gen Psychiatry. 1994;51(7):552–8. doi: 10.1001/archpsyc.1994.03950070044009 8031228

[11] Akdag SJ, Nestor PG, O’Donnell BF, Niznikiewicz MA, Shenton ME, McCarley RW. The startle reflex in schizophrenia: habituation and personality correlates. Schizophr Res. 2003;64(2–3):165–73. 14613681 10.1016/s0920-9964(03)00059-8 PMC2845846

[12] De Luca MA. Habituation of the responsiveness of mesolimbic and mesocortical dopamine transmission to taste stimuli. Front Integr Neurosci. 2014;8:21. doi: 10.3389/fnint.2014.00021 24624065 PMC3941202

[13] Aschan G. Habituation to repeated rotatory stimuli (cupulometry) and the effect of antinausea drugs and alcohol on the results. Acta Otolaryngol. 1967;64(2):95–106. doi: 10.3109/00016486709139096 4861759

[14] Ingle D. Reduction of habituation of prey-catching activity by alcohol intoxication in the frog. Behav Biol. 1973;8(1):123–9. doi: 10.1016/s0091-6773(73)80013-6 4540292

[15] Peeke HV, Peeke SC, Avis HH, Ellman G. Alcohol, habituation and the patterning of aggressive responses in a cichlid fish. Pharmacol Biochem Behav. 1975;3(6):1031–6. doi: 10.1016/0091-3057(75)90012-x 1241444

[16] Rinaldi PC, Nishimura LY, Thompson RF. Acute ethanol treatment modifies response properties and habituation of the DR-VR reflex in the isolated frog spinal cord. Alcohol Clin Exp Res. 1983;7(2):194–8. doi: 10.1111/j.1530-0277.1983.tb05438.x 6346923

[17] Lister RG. The effects of repeated doses of ethanol on exploration and its habituation. Psychopharmacology (Berl). 1987;92(1):78–83. doi: 10.1007/BF00215483 3110832

[18] Berthoz A, Young L, Oliveras F. Action of alcohol on vestibular compensation and habituation in the cat. Acta Otolaryngol. 1977;84(5–6):317–27. doi: 10.3109/00016487709123973 303424

[19] Glanzman DL, Epperlein RC. Disruption of vertebrate monosynaptic habituation by ethyl alcohol. Brain Res. 1981;212(1):117–26. doi: 10.1016/0006-8993(81)90037-8 6261882

[20] Dinges D, Kribbs N. Comparison of the effects of alcohol and sleepiness on simple reaction time performance: enhanced habituation as a common process. Alcohol Drugs Driv. 1989;5(4):329–39.

[21] Rankin CH, Beck CD, Chiba CM. Caenorhabditis elegans: a new model system for the study of learning and memory. Behav Brain Res. 1990;37(1):89–92. 2310497 10.1016/0166-4328(90)90074-o

[22] Rankin CH, Wicks SR. Mutations of the caenorhabditis elegans brain-specific inorganic phosphate transporter eat-4 affect habituation of the tap-withdrawal response without affecting the response itself. J Neurosci Off J Soc Neurosci. 2000 Jun 1;20(11):4337–44. 10.1523/JNEUROSCI.20-11-04337.2000 PMC6772661 10818169

[23] Sanyal S, Wintle RF, Kindt KS, Nuttley WM, Arvan R, Fitzmaurice P, et al. Dopamine modulates the plasticity of mechanosensory responses in Caenorhabditis elegans. EMBO J. 2004;23(2):473–82. doi: 10.1038/sj.emboj.7600057 14739932 PMC1271763

[24] Lee J, Jee C, McIntire SL. Ethanol preference in C. elegans. Genes Brain Behav. 2009;8(6):578–85. doi: 10.1111/j.1601-183X.2009.00513.x 19614755 PMC2880621

[25] Swierczek NA, Giles AC, Rankin CH, Kerr RA. High-throughput behavioral analysis in C. elegans. Nat Methods. 2011;8(7):592–8. doi: 10.1038/nmeth.1625 21642964 PMC3128206

[26] Giles AC, Rankin CH. Behavioral and genetic characterization of habituation using Caenorhabditis elegans. Neurobiol Learn Mem. 2009;92(2):139–46. doi: 10.1016/j.nlm.2008.08.004 18771741

[27] McDiarmid TA, Belmadani M, Liang J, Meili F, Mathews EA, Mullen GP, et al. Systematic phenomics analysis of autism-associated genes reveals parallel networks underlying reversible impairments in habituation. Proc Natl Acad Sci U S A. 2020;117(1):656–67. doi: 10.1073/pnas.1912049116 31754030 PMC6968627

[28] Morgan PG, Sedensky MM. Mutations affecting sensitivity to ethanol in the nematode, Caenorhabditis elegans. Alcohol Clin Exp Res. 1995;19(6):1423–9. doi: 10.1111/j.1530-0277.1995.tb01002.x 8749805

[29] Davies AG, Pierce-Shimomura JT, Kim H, VanHoven MK, Thiele TR, Bonci A, et al. A central role of the BK potassium channel in behavioral responses to ethanol in C. elegans. Cell. 2003;115(6):655–66. doi: 10.1016/s0092-8674(03)00979-6 14675531

[30] Davies A, Bettinger J, Thiele T, Judy M, McIntire S. Natural variation in the npr-1 gene modifies ethanol responses of wild strains of C. elegans. Neuron. 2004;42(5):731–43. 15182714 10.1016/j.neuron.2004.05.004

[31] Mitchell P, Mould R, Dillon J, Glautier S, Andrianakis I, James C, et al. A differential role for neuropeptides in acute and chronic adaptive responses to alcohol: behavioural and genetic analysis in Caenorhabditis elegans. PLoS One. 2010;5(5):e10422. doi: 10.1371/journal.pone.0010422 20454655 PMC2862703

[32] Speca DJ, Chihara D, Ashique AM, Bowers MS, Pierce-Shimomura JT, Lee J, et al. Conserved role of unc-79 in ethanol responses in lightweight mutant mice. PLoS Genet. 2010;6(8):e1001057. doi: 10.1371/journal.pgen.1001057 20714347 PMC2920847

[33] Alaimo JT, Davis SJ, Song SS, Burnette CR, Grotewiel M, Shelton KL, et al. Ethanol metabolism and osmolarity modify behavioral responses to ethanol in C. elegans. Alcohol Clin Exp Res. 2012;36(11):1840–50. doi: 10.1111/j.1530-0277.2012.01799.x 22486589 PMC3396773

[34] Jee C, Lee J, Lim JP, Parry D, Messing RO, McIntire SL. SEB-3, a CRF receptor-like GPCR, regulates locomotor activity states, stress responses and ethanol tolerance in Caenorhabditis elegans. Genes Brain Behav. 2013;12(2):250–62. doi: 10.1111/j.1601-183X.2012.00829.x 22853648 PMC3848202

[35] Raabe RC, Mathies LD, Davies AG, Bettinger JC. The omega-3 fatty acid eicosapentaenoic acid is required for normal alcohol response behaviors in C. elegans. PLoS One. 2014;9(8):e105999. doi: 10.1371/journal.pone.0105999 25162400 PMC4146551

[36] Katner SN, Bredhold KE, Steagall KB 2nd, Bell RL, Neal-Beliveau BS, Cheong MC, et al. Caenorhabditis elegans as a model system to identify therapeutics for alcohol use disorders. Behav Brain Res. 2019;365:7–16. doi: 10.1016/j.bbr.2019.02.015 30802531 PMC6474658

[37] Mathies LD, Blackwell GG, Austin MK, Edwards AC, Riley BP, Davies AG, et al. SWI/SNF chromatin remodeling regulates alcohol response behaviors in Caenorhabditis elegans and is associated with alcohol dependence in humans. Proc Natl Acad Sci U S A. 2015;112(10):3032–7. doi: 10.1073/pnas.1413451112 25713357 PMC4364201

[38] Scott LL, Davis SJ, Yen RC, Ordemann GJ, Nordquist SK, Bannai D, et al. Behavioral Deficits Following Withdrawal from Chronic Ethanol Are Influenced by SLO Channel Function in Caenorhabditis elegans. Genetics. 2017;206(3):1445–58. doi: 10.1534/genetics.116.193102 28546434 PMC5500142

[39] Wu ZQ, Li K, Ma JK, Li ZJ. Effects of ethanol intake on anti-oxidant responses and the lifespan of Caenorhabditis elegans. CyTA J Food. 2019;17(1):288–96.

[40] Pandey P, Singh A, Kaur H, Ghosh-Roy A, Babu K. Increased dopaminergic neurotransmission results in ethanol dependent sedative behaviors in Caenorhabditis elegans. PLoS Genet. 2021;17(2):e1009346. doi: 10.1371/journal.pgen.1009346 33524034 PMC7877767

[41] Albrecht PA, Fernandez-Hubeid LE, Deza-Ponzio R, Martins AC, Aschner M, Virgolini MB. Developmental lead exposure affects dopaminergic neuron morphology and modifies basal slowing response in Caenorhabditis elegans: effects of ethanol. Neurotoxicology. 2022;91:349–59. doi: 10.1016/j.neuro.2022.06.005 35724878

[42] Guzman DM, Chakka K, Shi T, Marron A, Fiorito AE, Rahman NS, et al. Transgenerational effects of alcohol on behavioral sensitivity to alcohol in Caenorhabditis elegans. PLoS One. 2022;17(10):e0271849. doi: 10.1371/journal.pone.0271849 36256641 PMC9578632

[43] Salim C, Kan AK, Batsaikhan E, Patterson EC, Jee C. Neuropeptidergic regulation of compulsive ethanol seeking in C. elegans. Sci Rep. 2022;12(1):1804. 35110557 10.1038/s41598-022-05256-1 PMC8810865

[44] Dhawan R, Dusenbery D, Williams P. Comparison of lethality, reproduction, and behavior as toxicological endpoints in the nematode Caenorhabditis elegans. J Toxicol Environ Health A. 1999;58(7):451–62. 10616193 10.1080/009841099157179

[45] Johnson JR, Edwards MR, Davies H, Newman D, Holden W, Jenkins RE, et al. Ethanol Stimulates Locomotion via a Gαs-Signaling Pathway in IL2 Neurons in Caenorhabditis elegans. Genetics. 2017;207(3):1023–39. doi: 10.1534/genetics.117.300119 28951527 PMC5676223

[46] Hawkins EG, Martin I, Kondo LM, Judy ME, Brings VE, Chan C-L, et al. A novel cholinergic action of alcohol and the development of tolerance to that effect in Caenorhabditis elegans. Genetics. 2015;199(1):135–49. doi: 10.1534/genetics.114.171884 25342716 PMC4286678

[47] Bettinger JC, McIntire SL. State-dependency in C. elegans. Genes Brain Behav. 2004;3(5):266–72. doi: 10.1111/j.1601-183X.2004.00080.x 15344920

[48] Dopico AM, Anantharam V, Treistman SN. Ethanol increases the activity of Ca(++)-dependent K+ (mslo) channels: functional interaction with cytosolic Ca++. J Pharmacol Exp Ther. 1998;284(1):258–68. doi: 10.1016/s0022-3565(24)37207-6 9435186

[49] Bukiya AN, McMillan JE, Fedinec AL, Patil SA, Miller DD, Leffler CW, et al. Cerebrovascular dilation via selective targeting of the cholane steroid-recognition site in the BK channel β1-subunit by a novel nonsteroidal agent. Mol Pharmacol. 2013;83(5):1030–44. doi: 10.1124/mol.112.083519 23455312 PMC3629834

[50] Bukiya AN, Kuntamallappanavar G, Edwards J, Singh AK, Shivakumar B, Dopico AM. An alcohol-sensing site in the calcium- and voltage-gated, large conductance potassium (BK) channel. Proc Natl Acad Sci U S A. 2014;111(25):9313–8. 24927535 10.1073/pnas.1317363111 PMC4078796

[51] Davis SJ, Scott LL, Ordemann G, Philpo A, Cohn J, Pierce-Shimomura JT. Putative calcium-binding domains of the Caenorhabditis elegans BK channel are dispensable for intoxication and ethanol activation. Genes Brain Behav. 2015;14(6):454–65. doi: 10.1111/gbb.12229 26113050 PMC4885643

[52] Davis SJ, Scott LL, Hu K, Pierce-Shimomura JT. Conserved single residue in the BK potassium channel required for activation by alcohol and intoxication in C. elegans. J Neurosci. 2014;34(29):9562–73. 25031399 10.1523/JNEUROSCI.0838-14.2014 PMC4099540

[53] Liu J, Vaithianathan T, Manivannan K, Parrill A, Dopico AM. Ethanol modulates BKCa channels by acting as an adjuvant of calcium. Mol Pharmacol. 2008 Sep;74(3):628–40. 18552122 10.1124/mol.108.048694 PMC2764333

[54] Yuan P, Leonetti MD, Pico AR, Hsiung Y, MacKinnon R. Structure of the human BK channel Ca2+-activation apparatus at 3.0 A resolution. Science. 2010;329(5988):182–6. doi: 10.1126/science.1190414 20508092 PMC3022345

[55] Brenner S. The genetics of Caenorhabditis elegans. Genetics. 1974;77(1):71–94. doi: 10.1093/genetics/77.1.71 4366476 PMC1213120

[56] Oh K, Haney J, Wang X, Chuang C, Richmond J, Kim H. Erg-28 controls bk channel trafficking in the er to regulate synaptic function and alcohol response in C. elegans. eLife. 2017;6:e24733. 10.7554/eLife.24733 PMC5295816 28168949

[57] Stiernagle T. Maintenance of C. elegans. WormBook [Internet]. 2006 [cited 2024 Sep 4]; Available from: http://www.wormbook.org/chapters/www_strainmaintain/strainmaintain.html 10.1895/wormbook.1.101.1 PMC4781397 18050451

[58] Altun ZF, Hall DH. Introduction. [cited 2024 Sep 4]; Available from: http://www.wormatlas.org/hermaphrodite/introduction/Introframeset.html

[59] Wicks SR, Rankin CH. Recovery from habituation in Caenorhabditis elegans is dependent on interstimulus interval and not habituation kinetics. Behav Neurosci. 1996;110(4):840–4. doi: 10.1037//0735-7044.110.4.840 8864275

[60] McEwan A. Modulation of habituation kinetics and behavioural shifts by members of the heterotrimeric G-protein signaling pathways [Internet]. University of British Columbia; 2013 [cited 2024 Sep 4]. Available from: https://open.library.ubc.ca/soa/cIRcle/collections/ubctheses/24/items/1.0073828

[61] Hong RL, Svatos A, Herrmann M, Sommer RJ. Species-specific recognition of beetle cues by the nematode Pristionchus maupasi. Evol Dev. 2008;10(3):273–9. 18460089 10.1111/j.1525-142X.2008.00236.x

[62] Martin GE. BK channel and alcohol, a complicated affair. Int Rev Neurobiol. 2010;91:321–38. doi: 10.1016/S0074-7742(10)91010-6 20813247

[63] Bettinger JC, Leung K, Bolling MH, Goldsmith AD, Davies AG. Lipid environment modulates the development of acute tolerance to ethanol in Caenorhabditis elegans. PLoS One. 2012;7(5):e35192. doi: 10.1371/journal.pone.0035192 22574115 PMC3344825

[64] Dillon J, Andrianakis I, Mould R, Ient B, Liu W, James C, et al. Distinct molecular targets including SLO-1 and gap junctions are engaged across a continuum of ethanol concentrations in Caenorhabditis elegans. FASEB J. 2013;27(10):4266–78. doi: 10.1096/fj.11-189340 23882127 PMC4046189

[65] Dopico AM, Bukiya AN, Martin GE. Ethanol modulation of mammalian BK channels in excitable tissues: molecular targets and their possible contribution to alcohol-induced altered behavior. Front Physiol. 2014;5:466. doi: 10.3389/fphys.2014.00466 25538625 PMC4256990

[66] Wang Z, Saifee O, Nonet M, Salkoff L. Slo-1 potassium channels control quantal content of neurotransmitter release at the C. elegans neuromuscular junction. Neuron. 2001;32(5):867–81. 11738032 10.1016/s0896-6273(01)00522-0

[67] Davis P, Zarowiecki M, Arnaboldi V, Becerra A, Cain S, Chan J, et al. WormBase in 2022-data, processes, and tools for analyzing Caenorhabditis elegans. Genetics. 2022;220(4):iyac003. doi: 10.1093/genetics/iyac003 35134929 PMC8982018

[68] Wang S, Tang NH, Lara-Gonzalez P, Zhao Z, Cheerambathur DK, Prevo B, et al. A toolkit for GFP-mediated tissue-specific protein degradation in C. elegans. Development. 2017;144(14):2694–701. doi: 10.1242/dev.150094 28619826 PMC5536931

[69] Hernández OH, García-Martínez R, Monteón V. Alcohol effects on the P2 component of auditory evoked potentials. An Acad Bras Cienc. 2014;86(1):437–49. 24519012 10.1590/0001-3765201420130301

[70] Kang D, Bresin K, Fairbairn CE. The impact of alcohol and social context on the startle eyeblink reflex. Alcohol Clin Exp Res. 2018;42(10):1951–60. doi: 10.1111/acer.13838 29989675 PMC6167173

[71] Traynor ME, Woodson PB, Schlapfer WT, Barondes SH. Tolerance to an effect of ethanol on post-tetanic potentiation in Aplysia. Drug Alcohol Depend. 1977;2(5–6):361–70. doi: 10.1016/0376-8716(77)90038-2 913239

[72] Davies AG, Blackwell GG, Raabe RC, Bettinger JC. An assay for measuring the effects of ethanol on the locomotion speed of Caenorhabditis elegans. J Vis Exp. 2015;98:52681. 10.3791/52681 PMC4476067 25938273

[73] Faumont S, Lindsay TH, Lockery SR. Neuronal microcircuits for decision making in C. elegans. Curr Opin Neurobiol. 2012;22(4):580–91. doi: 10.1016/j.conb.2012.05.005 22699037 PMC3593597

[74] Wicks SR, Rankin CH. Integration of mechanosensory stimuli in Caenorhabditis elegans. J Neurosci. 1995;15(3 Pt 2):2434–44. doi: 10.1523/JNEUROSCI.15-03-02434.1995 7891178 PMC6578104

[75] Croll NA. Components and patterns in the behaviour of the nematode Caenorhabditis elegans. J Zool. 1975;176(2):159–76.

[76] Rose JK, Rankin CH. Analyses of habituation in Caenorhabditis elegans. Learn Mem. 2001;8(2):63–9. doi: 10.1101/lm.37801 11274251

[77] Roberts W, Augustine S, Lawton K, Lindsay T, Thiele T, Izquierdo E. A stochastic neuronal model predicts random search behaviors at multiple spatial scales in C. elegans. eLife. 2016;5:e12572. 10.7554/eLife.12572 PMC4798983 26824391

[78] Chalfie M, Sulston JE, White JG, Southgate E, Thomson JN, Brenner S. The neural circuit for touch sensitivity in Caenorhabditis elegans. J Neurosci. 1985;5(4):956–64. doi: 10.1523/JNEUROSCI.05-04-00956.1985 3981252 PMC6565008

[79] Wicks SR, Roehrig CJ, Rankin CH. A dynamic network simulation of the nematode tap withdrawal circuit: predictions concerning synaptic function using behavioral criteria. J Neurosci. 1996;16(12):4017–31. doi: 10.1523/JNEUROSCI.16-12-04017.1996 8656295 PMC6578605

[80] Salkoff L, Butler A, Ferreira G, Santi C, Wei A. High-conductance potassium channels of the SLO family. Nat Rev Neurosci. 2006;7(12):921–31. 17115074 10.1038/nrn1992

[81] Piggott BJ, Liu J, Feng Z, Wescott SA, Xu XZS. The neural circuits and synaptic mechanisms underlying motor initiation in C. elegans. Cell. 2011;147(4):922–33. doi: 10.1016/j.cell.2011.08.053 22078887 PMC3233480

