# Supplementary material for: Ethanol alters mechanosensory habituation in C. elegans by way of the BK potassium channel through a novel mechanism
Source: PLoS One. 2025 Jun 11;20(6):e0315069. doi: 10.1371/journal.pone.0315069 (PMC12157217; doi:10.1371/journal.pone.0315069)
Supplement: S1 File — (ZIP) [file pone.0315069.s001.zip › slo-1 supplemental figures/slo-1 supplemental figures.docx]

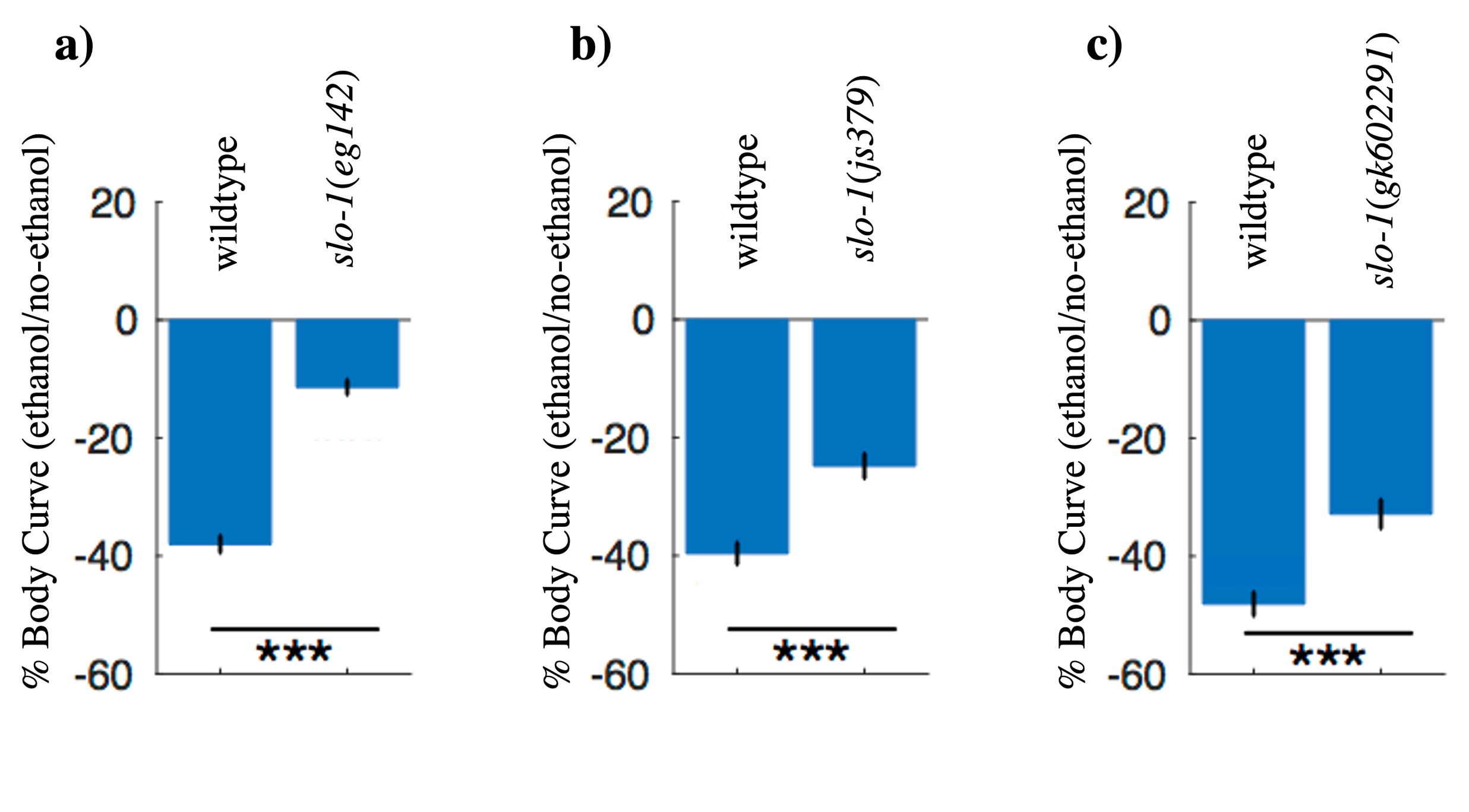


**Figure S1: Null mutations and the T381I point mutation in *slo-1* reduce the effect of 400 mM ethanol on body curve.**

(**a**) *slo-1*(*eg142*) mutants displayed significantly less loss of body curve on 400 mM ethanol than wildtype animals. Body curve data was taken from 5-10s preceding the first tap stimulus. % body curve was calculated by dividing the mean body curve of a 400 mM plate by the mean of body curve of all 0 mM plates within the same experiment (**b**) *slo-1*(*js379*) mutants displayed significantly less loss of body curve on 400 mM ethanol than wildtype animals. (**c**) *slo-1*(*gk602291*) mutants displayed significantly less loss of body curve on 400 mM ethanol than wildtype animals. *** = p < .001.


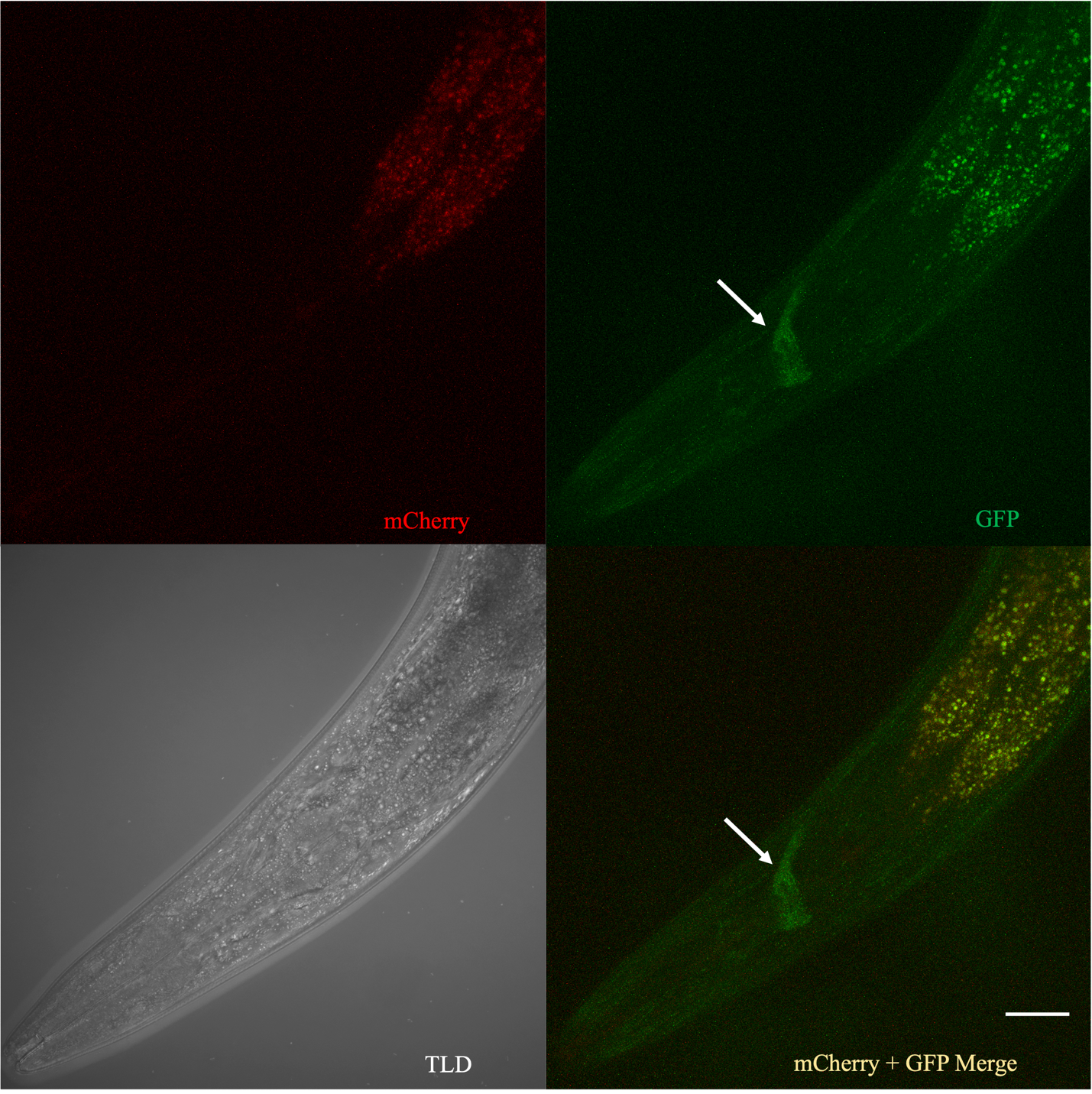


**Figure S2: Anterior expression pattern of *slo-1* in a *slo-1::gfp* worm.**

These four panels display a 2D representation of a z-stack taken of the head of a *slo-1::gfp* worm. (**bottom-left**) Image of the head of the animal illuminated with white light. (**top-left**) As a control, no red fluorescence was observed in the nerve ring. (**top-right**) *slo-1* expression is observed as green fluorescence band in the nerve ring (white arrows). (**bottom-right**) Composite of the red and green fluorescent images. Yellow fluorescence is observed when both the red and green fluorescence are superimposed. Yellow fluorescence was observed in autofluorescence in the gut. N=1. A 543 nm laser was used for red fluorescence, and a 488 nm laser was used for green fluorescence. Scale bar = 25 μm.


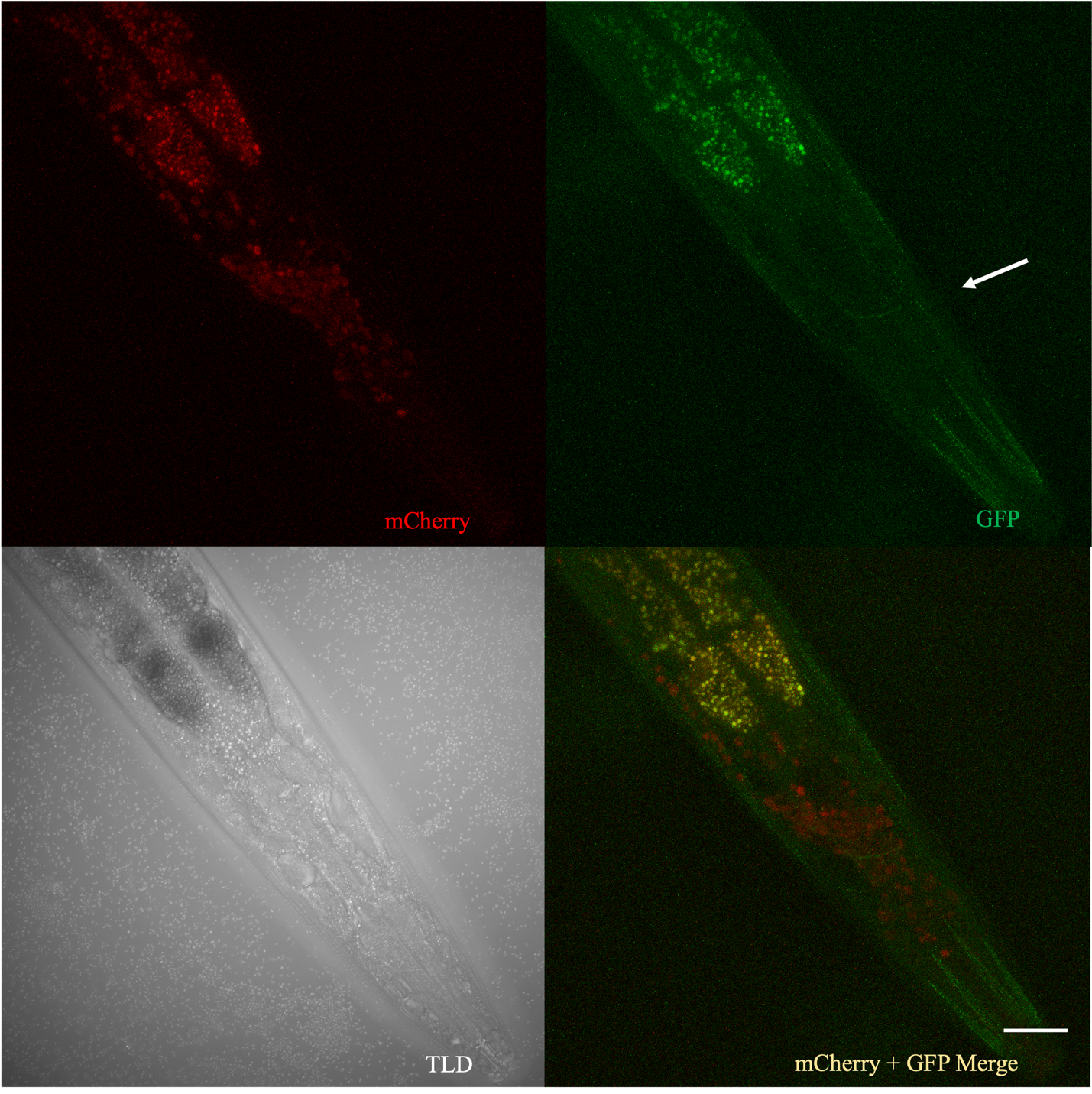


**Figure S3: *slo-1::gfp* expression in the nerve ring is lost with pan-neuronal expression of the GFP degron.**

These four panels display a 2D representation of a z-stack taken of the head of a representative HKK1165 worm containing *slo-1::gfp* and the GFP degron expressed pan-neuronally. (**bottom-left**) Image of the head of the animal illuminated with white light. (**top-left**) mCherry is expressed pan-neuronally, illuminating the many neurons in the head. (**top-right**) The band of green fluorescence observed in the nerve ring of *slo-1::gfp* animals was not present in these animals (white arrows). (**bottom-right**) Composite of the red and green fluorescent images. Yellow fluorescence is observed when both the red and green fluorescence are superimposed. The only yellow fluorescence observed was autofluorescence in the gut. This indicates that SLO-1::GFP is being efficiently degraded in the nerve ring. Genotype: cim105[slo-1::GFP]; cimSi1[rgef-1p::vhhGFP::zif-1::operon-linker::mCherry::his-11::tbb-2 3’UTR+cbr-unc-119(+)])). N=3. A 543 nm laser was used for red fluorescence, and a 488 nm laser was used for green fluorescence. Scale bar = 25 μm.


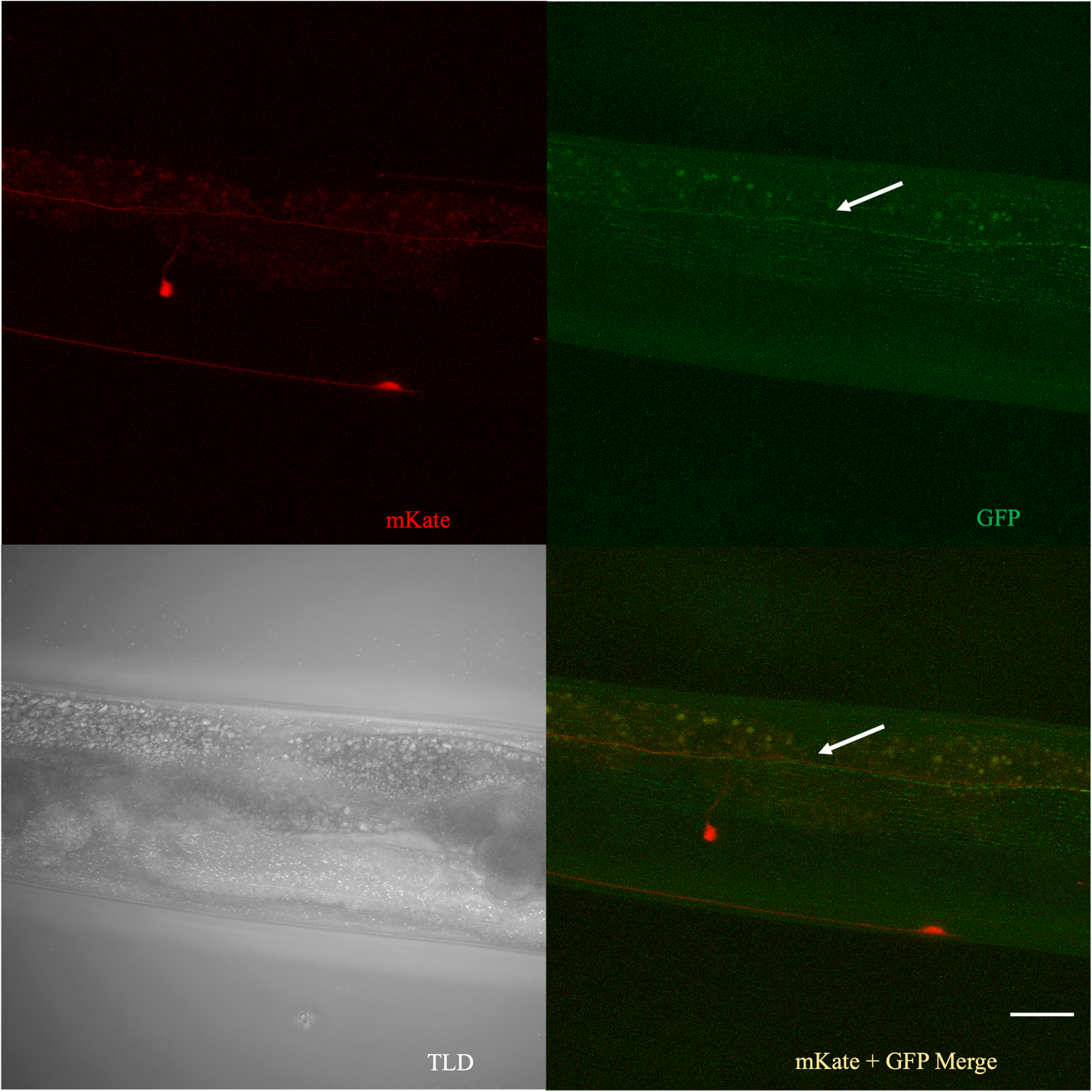


**Figure S4: *slo-1::gfp* expression is not visible in AVM and ALM with mechanosensory expression of the GFP degron.**

These four panels display a 2D representation of a z-stack taken of the anterior midsection of a representative VG903 worm containing *slo-1::gfp* and the GFP degron expressed in mechanosensory neurons. (**bottom-left**) Image of the anterior midsection of the animal illuminated with white light. (**top-left**) mKate fills the cell body and processes of AVM (left cell body) and at least one ALM (right cell body). (**top-right**) Only a line of *slo-1::gfp* fluorescence is observed in the ventral nerve cord (white arrow), indicating expression of *slo-1::gfp* in other neurons but not in AVM and ALM. (**bottom-right**) Composite of the red and green fluorescent images. Yellow fluorescence is observed when both the red and green fluorescence are superimposed. The only yellow fluorescence observed was autofluorescence in the gut. No overlap of slo-1::gfp and mKate is observed in AVM or ALM, even in the ventral nerve cord (white arrow), the slo-1::gfp and the mKate filled AVM processes do not overlap. This indicates that SLO-1::GFP is being efficiently degraded in these mechanosensory neurons. Genotype: cim105[slo-1::GFP]; ltsi953[mec-18p::vhhGFP4::zif-1::operon-linker::mKate::his-11::tbb-2 3’UTR+cbr-unc-119(+)])). N=2. A 543 nm laser was used for red fluorescence, and a 488 nm laser was used for green fluorescence. Scale bar = 25 μm.


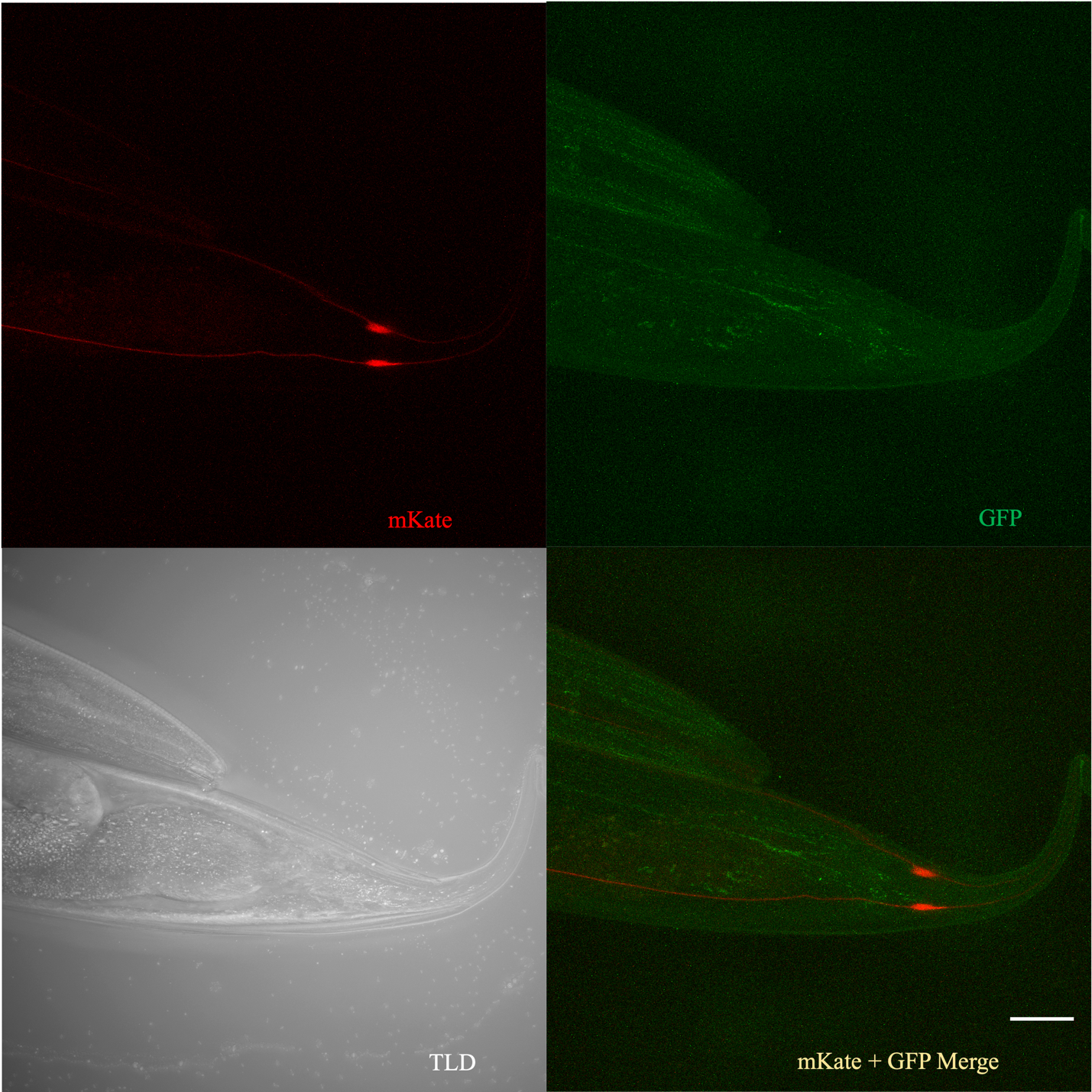


**Figure S5: *slo-1::gfp* expression is not visible in PLML and PLMR with mechanosensory expression of the GFP degron.**

These four panels display a 2D representation of a z-stack taken of the tail of a representative VG903 worm containing *slo-1::gfp* and the GFP degron expressed in mechanosensory neurons. (**bottom-left**) Image of the tail of the animal illuminated with white light. (**top-left**) mKate fills the cell body and processes of the PLM neurons. (**top-right**) *slo-1::gfp* expression in the tail after degradation in mechanosensory neurons. (**bottom-right**) Composite of the red and green fluorescent images. Yellow fluorescence is observed when both the red and green fluorescence are superimposed. No overlap of slo-1::gfp and mKate is observed in the PLM neuron cell bodies, or their processes. This indicates that SLO-1::GFP is being efficiently degraded in these mechanosensory neurons. Genotype: cim105[slo-1::GFP]; ltsi953[mec-18p::vhhGFP4::zif-1::operon-linker::mKate::his-11::tbb-2 3’UTR+cbr-unc-119(+)])). N=1. A 543 nm laser was used for red fluorescence, and a 488 nm laser was used for green fluorescence. Scale bar = 25 μm.


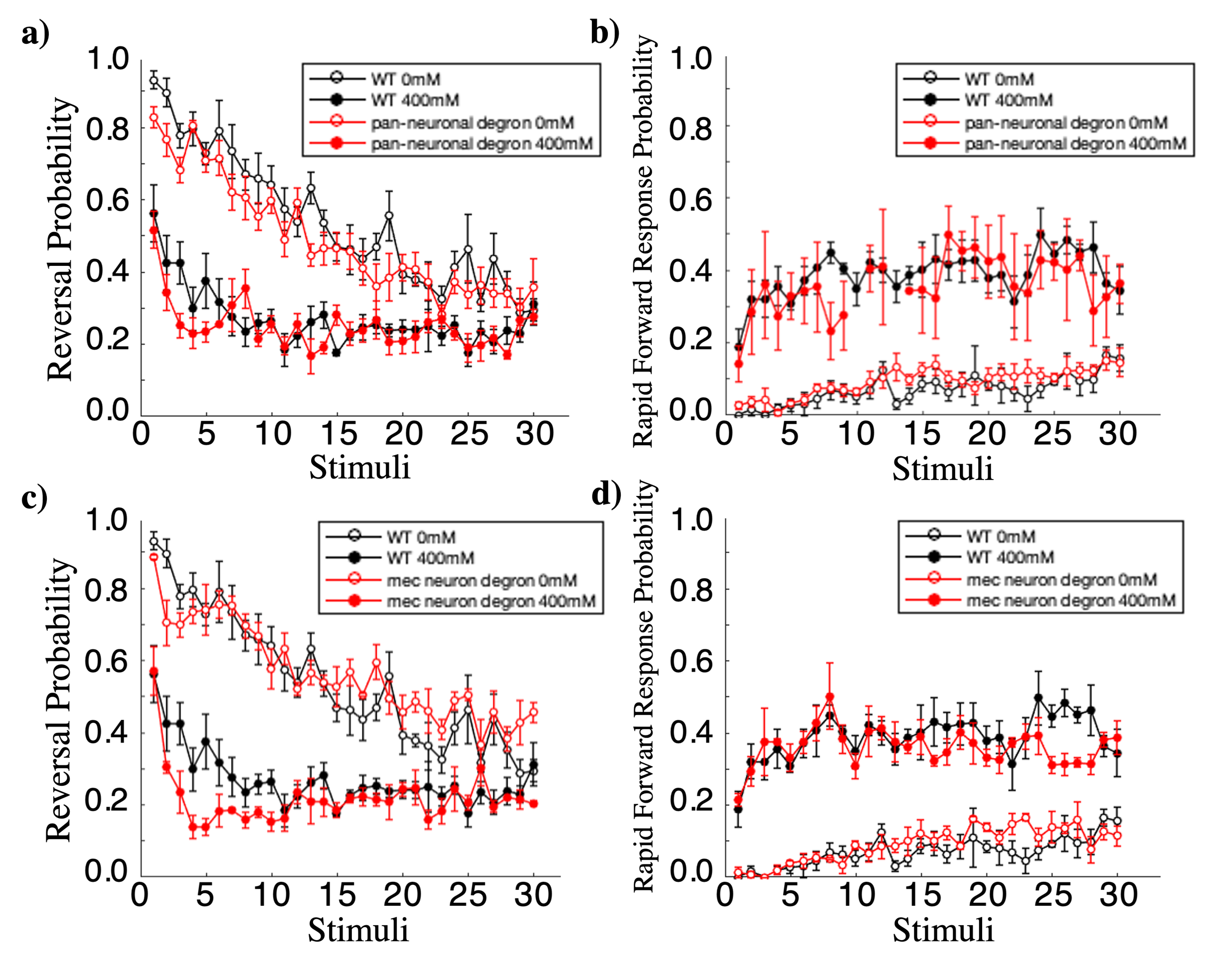


**Figure S6: GFP degron expression pan-neuronally, or just in the mechanosensory neurons, did not impact reversal or rapid forward response probability, or the effect of 400 mM ethanol on response probability.**

(**a**) Ethanol has a similar effect on reversal and (**b**) rapid forward response probability in worms expressing the GFP degron pan-neuronally as it does in wild-type worms. (**c**) Ethanol has a similar effect on reversal and (**d**) rapid forward response probability in worms expressing the GFP degron in mechanosensory neurons as it does in wild-type worms. Moreover, the degron expression did not impact response probability.
