# Supplementary figures and images for: Ethanol alters mechanosensory habituation in C. elegans by way of the BK potassium channel through a novel mechanism
Source: PLoS One. 2025 Jun 11;20(6):e0315069. doi: 10.1371/journal.pone.0315069 (PMC12157217; doi:10.1371/journal.pone.0315069)

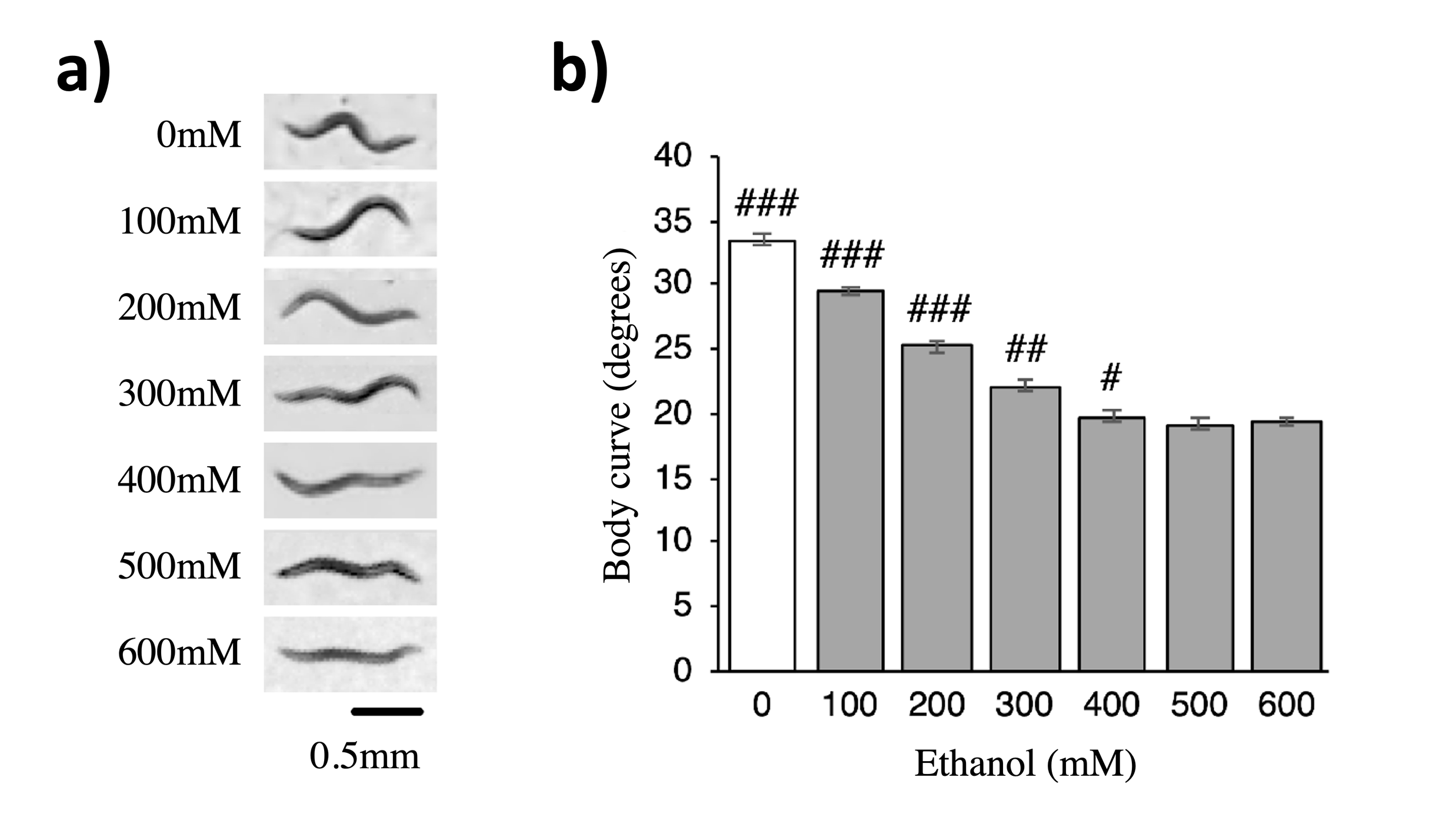

Supplement: S1 File — (ZIP) [file pone.0315069.s001.zip › slo-1 supplemental figures/slo-1_fig1.tif]

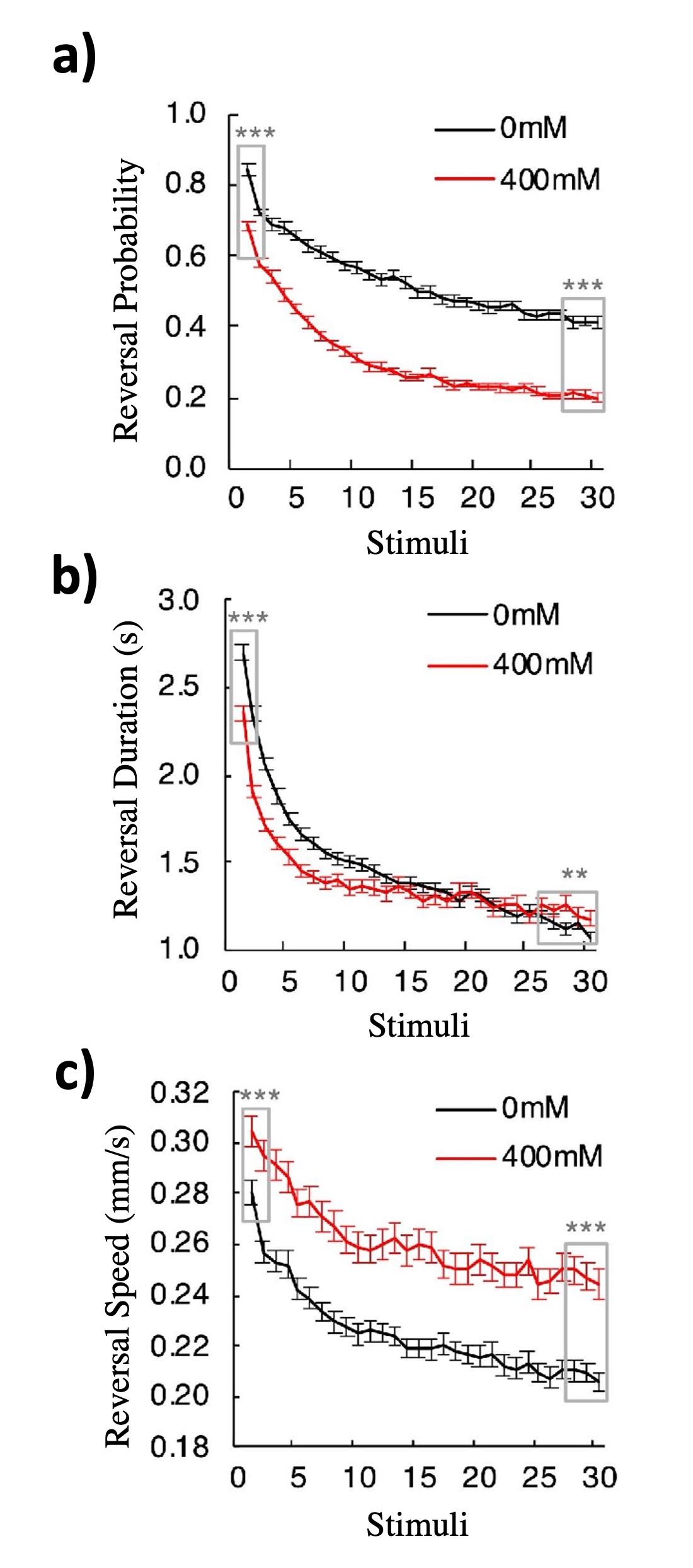

Supplement: S1 File — (ZIP) [file pone.0315069.s001.zip › slo-1 supplemental figures/slo-1_fig2.tif]

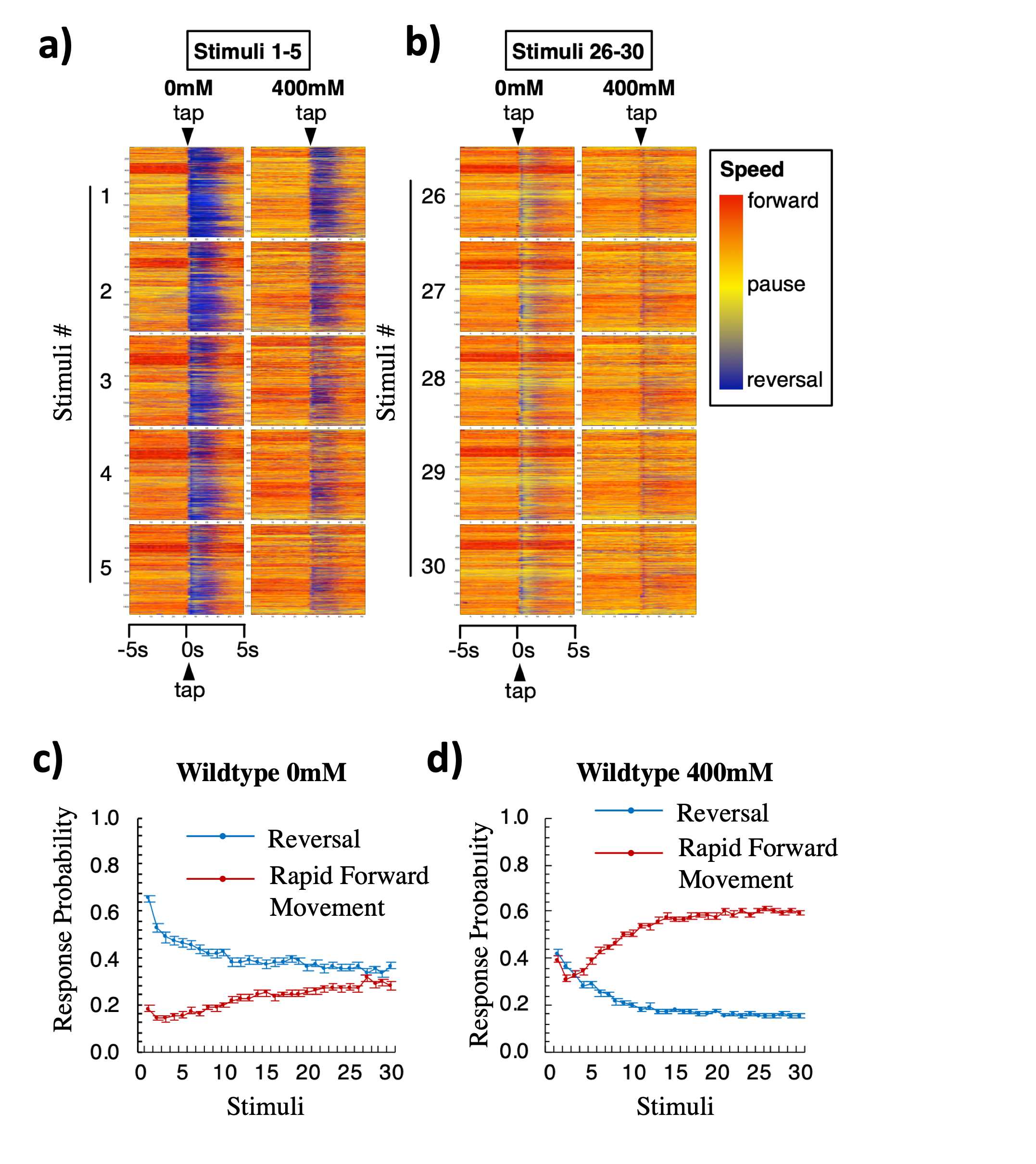

Supplement: S1 File — (ZIP) [file pone.0315069.s001.zip › slo-1 supplemental figures/slo-1_fig3.tif]

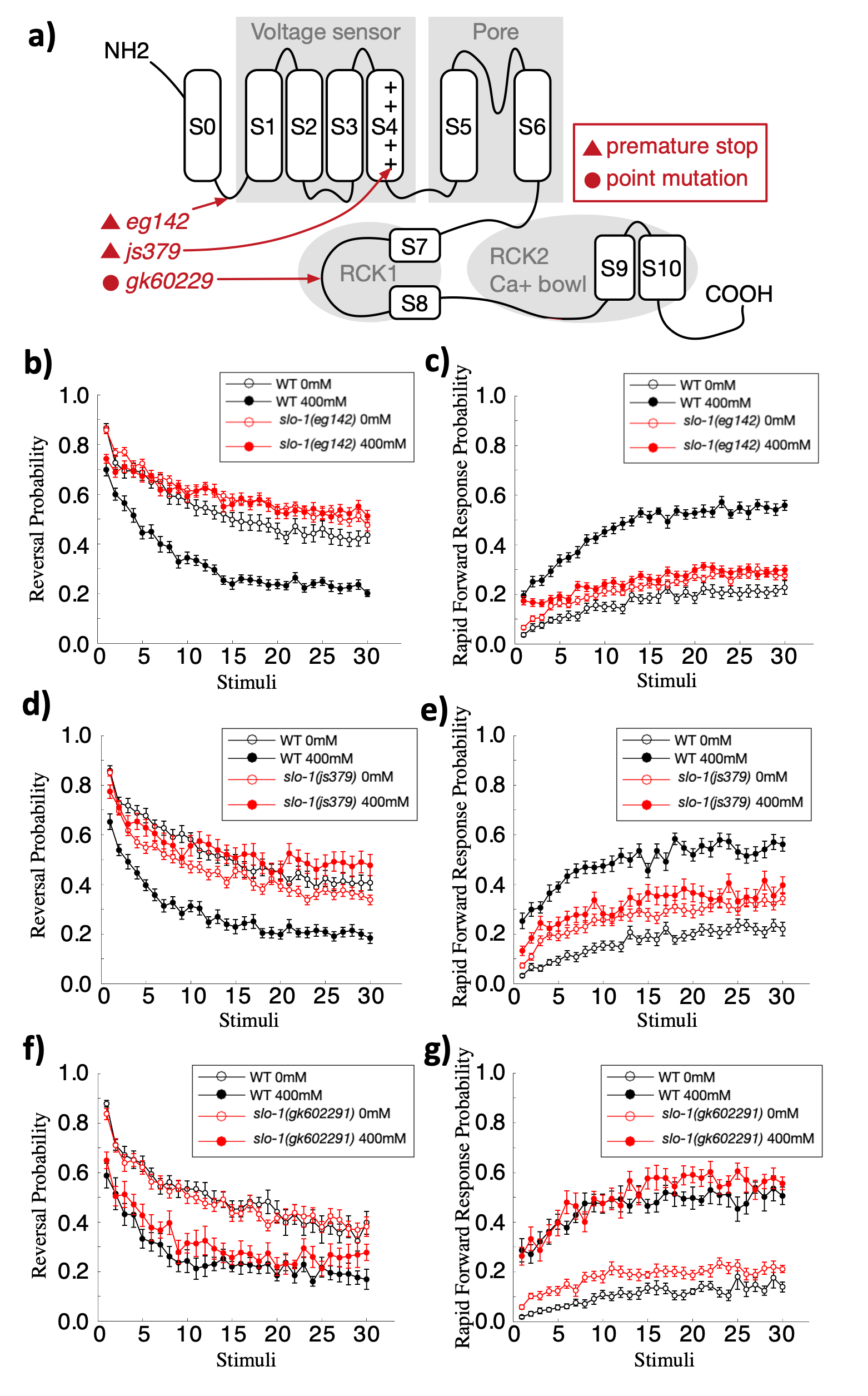

Supplement: S1 File — (ZIP) [file pone.0315069.s001.zip › slo-1 supplemental figures/slo-1_fig4.tif]

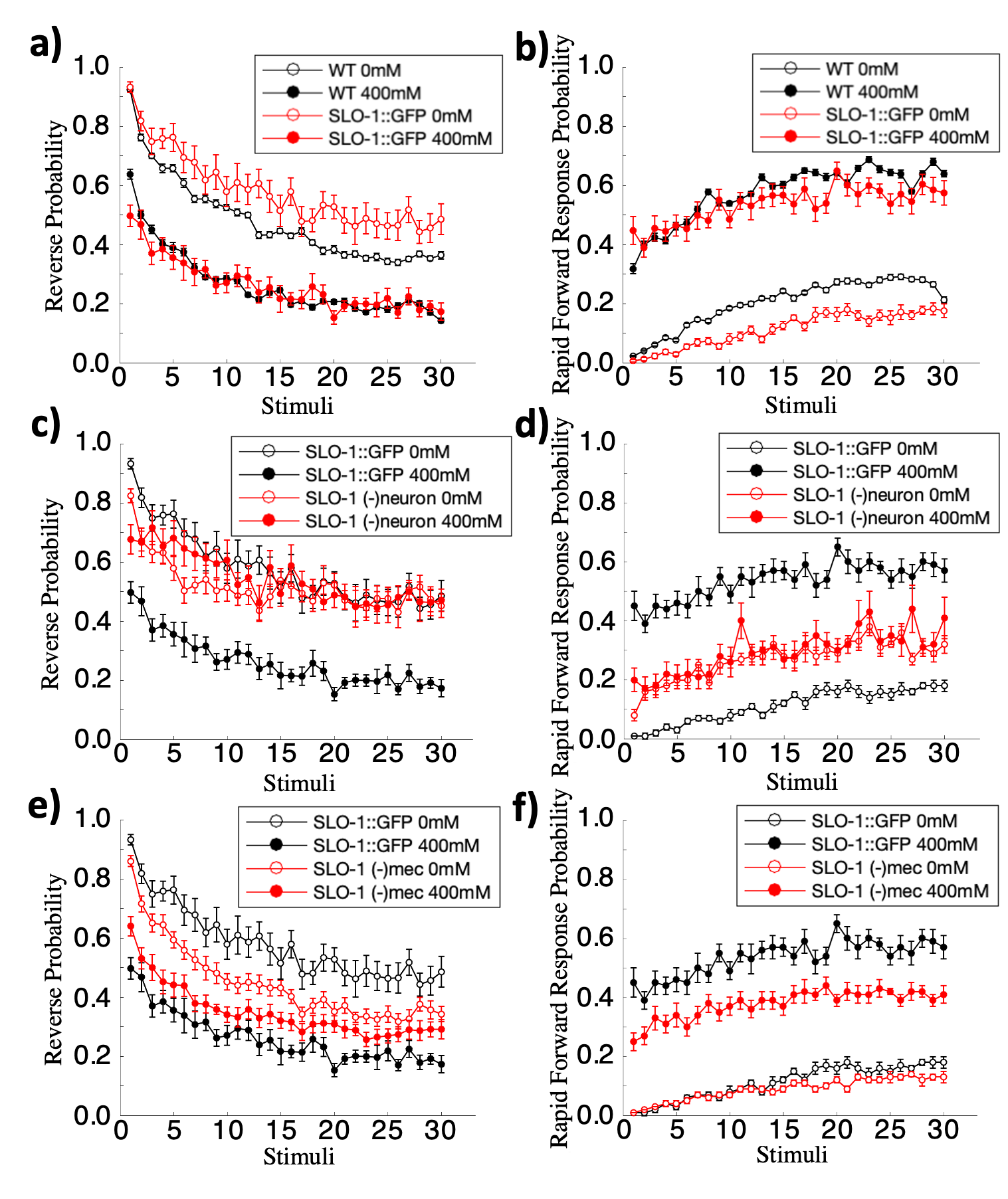

Supplement: S1 File — (ZIP) [file pone.0315069.s001.zip › slo-1 supplemental figures/slo-1_fig5.tif]
